# Modeling and analysis of UAV-Assisted sparse ground networks

**DOI:** 10.1371/journal.pone.0352585

**Published:** 2026-07-15

**Authors:** Huakui Sun, Yang Zhou

**Affiliations:** 1 School of Airspace Science and Engineering, Shandong University, Shandong, China; 2 School of Electronics and Information, Northwestern Polytechnical University, Xi’an, China; Northwestern Polytechnical University, CHINA

## Abstract

With the continuous improvement of communication requirements, it is difficult for traditional ground cellular networks to achieve seamless wide-area coverage, particularly in remote regions. To solve the insufficient coverage problem of sparse ground cellular network, we propose the Air-and-Ground Cooperative Network (AGCN) architecture to enhance the network coverage performance. Besides, and Cell Range Expansion (CRE) technology is combined to achieve load balancing between aerial Base Stations (BSs) and ground BSs. By setting user connection bias, communication users can access different BSs to coordinate the coverage performance of each layer in the AGCN. Based on stochastic geometry theory, we analyze the user access probability and distance distribution, and then derive the analytical formulas of network throughput and traversal rate. Furthermore, we analyze the influence of user connection bias on network performance in CRE technology. Simulation resulsts verify the correctness of the theoretial analysis and the efficiency of the AGCN in coverage enhancement. It indicates that there is an optimal bias setting to maximize the overall coverage performance of the AGCN, which provides valuable guidance for the future network design.

## Introduction

Although ground networks provide strong support for communication services, the explosive growth of communication data driven by diverse technological advances has rendered traditional ground networks incapable of satisfying the demands of global seamless wide-area coverage. Establishing efficient and reliable communication schemes in regions with sparse ground networks has thus emerged as a critical challenge. For scenarios requiring enhanced coverage, UAV-assisted network has become an effective solution. Compared with conventional ground networks, Unmanned Aerial Vehicle (UAV)-enabled networks offer superior communication links and coverage performance by virtue of their flexible positional deployment. As a key component of the air–ground integrated network, integrating UAVs into traditional terrestrial infrastructures to construct Air-and-Ground Cooperative Network (AGCN) has become an important research direction for 6G [1,2]. Employing UAVs as aerial Base Stations (BSs) enables dynamic adjustment of their height and location, thereby achieving high system throughput and reliable Line-of-Sight (LoS) coverage for ground user equipment [3,4].

In recent years, research and applications of UAVs have grown exponentially. UAVs are used as aerial relay for cell edge enhancement [5] and hotspot support [6] and aerial mobile edge node for semantic communications [7]. However, due to the distinct communication architectures between UAVs and terrestrial BSs, characterizing the performance of heterogeneous networks composed of aerial and ground nodes has become a significant challenge. To address this issue, stochastic geometry offers powerful mathematical tools for network modeling and analysis. With the evolution toward increasingly heterogeneous cellular networks, analytical results derived from single-tier network models are no longer sufficient to meet practical deployment requirements [8]. In [9], a *K*-tier heterogeneous network is investigated, where each tier is modeled as an independent Homogeneous Poisson Point Process (HPPP). Each tier is characterized by distinct transmit powers and spatial densities. The study derives the Signal-to-Interference-plus-Noise Ratio (SINR) coverage probability and average achievable rate, and further evaluates the performance of each tier under both open-access and closed-access strategies. In [10], a user-centric cellular network with UAV-assisted ground BSs is studied. In this model, UAVs are deployed at a fixed altitude, while users are spatially distributed around the ground projections of UAVs according to a Poisson Cluster Process (PCP). The impact of UAV altitude and path-loss exponent on network performance is analyzed.

Using the PPP framework, [11] examines UAV-assisted cellular networks in mixed urban, suburban, and rural environments. In this model, ground BSs follow a non-homogeneous PPP, whereas UAVs are modeled as an HPPP. Different service strategies are adopted across regions: urban areas are served by terrestrial BSs without UAV deployment, suburban areas rely on UAVs, and coverage characteristics vary across urban, suburban, and rural regions. In [12], UAVs are modeled as a Three-Dimensional (3D) HPPP, and spectrum sharing in UAV small cell networks is investigated, leading to the derivation of the optimal UAV deployment density. Furthermore, [13] analyzes the successful transmission probability and spectral efficiency of multi-layer aerial networks. For finite 3D UAV networks, [14] employs a Binomial Point Process (BPP) to model UAV locations and derives the downlink coverage probability. Although some studies adopt 3D point processes to model UAV deployments, such approaches assume that UAVs are randomly distributed in altitude, which significantly increases analytical complexity, especially when altitude is constrained. Therefore, to simplify analysis, most existing works still rely on two-dimensional (2D) point process models while assuming UAVs operate at a fixed altitude [15].

Due to the heterogeneity of modern cellular networks, offloading users or traffic across different tiers can effectively balance network performance and enhance overall Quality of Service (QoS). When significant disparities exist between Macro BSs (MBSs) and Small-cell BSs (SBS), Cell Range Expansion (CRE) can not only improve the average network throughput but also enhance the coverage performance of cell-edge users [15,16]. In [17], stochastic geometry is employed to analyze the downlink and uplink transmission performance of small cells in a two-tier heterogeneous network with CRE. The results indicate that there exists an optimal CRE bias for MBSs that maximizes network capacity. Furthermore, when this optimal bias is adopted, increasing the SBS density has a negligible impact on Energy Efficiency (EE), while significantly improving network throughput. In [18], the downlink performance of a two-tier heterogeneous network is investigated, where the spatial distribution of BSs is modeled using a HPPP. The results demonstrate that optimizing the CRE bias can substantially improve the network throughput. In addition, [19] proposes a low-complexity algorithm to optimize the CRE bias values. From the perspective of energy efficiency, [20] evaluates CRE performance and determines the optimal bias by constructing appropriate utility functions. In [21], an enhanced CRE scheme is proposed, where different bias values are assigned based on users’ association ratios with each Pico BS (PBS). Finally, [22] and [23] develop adaptive bias adjustment methods to further improve coverage performance.

Motivated by the above, we in this paper design and analyze the air-and-ground network cooperative networks. The main contributions are given as follows.

- We propose a new UAV-assisted sparse terrestrial network architecture. In this architecture, UAVs are deployed at a fixed altitude to enhance the coverage of the terrestrial network. CRE is further incorporated to balance the coverage performance between Aerial BSs (ABs) and Ground BSs (GBSs). By introducing adjustable bias settings, users can associate with different tiers of BSs, thereby alleviating the resource limitations of any single network layer.
- We analyze the performance of the UAV-assisted sparse terrestrial network. Accounting for the distinct transmission characteristics of ABSs and GBSs, we derive the general expressions for the user association probability, the distance distribution between users and their serving BSs, and the SINR coverage probability. Based on the derived SINR coverage probability, the network throughput density and rate density are further obtained.
- We conduct abundant simulations to validate our analysis and evaluate the network performance. We systematically analyze the impact of some key system parameters (SINR threshold, UAV height, Relative bias, UAV density, User density) on the network performance and discuss how to set these parameters. We find that a reasonable SINR threshold should be set to maximize the network throughput. Under different terrestrial network density, the optimal UAV height is different. Besides, It indicates that there is an optimal bias setting to maximize the overall coverage performance of the network.

The rest of this paper is organized as follows. In the System Model section, the UAV assisted sparse terrestrial network is described, in which the channel models of different transmission links are introduced in detail. On this basis, the corresponding performance indicators are introduced. In the Performance Analysis section, the communication performance of users accessing different types of BSs is analyzed including user access probability, distance distribution, network throughput, and traversal rate. The numerical simulation results and analysis are discussed in the Simulation Results section. Finally, the Conclusion section presents the conclusion and future work of the paper.

## System model

### Network model

**Fig 1:**
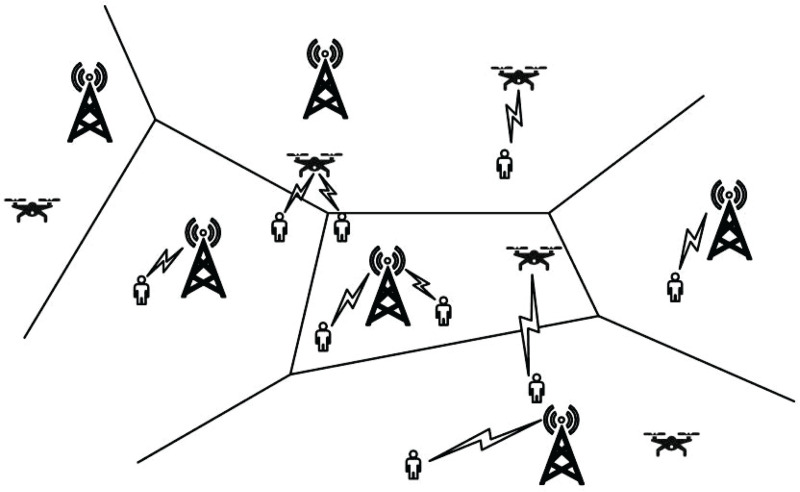
UAV-Assisted Sparse Ground Networks.

In this paper, we consider a UAV-assisted sparse terrestrial network, where the UAVs (act as ABSs) assist the GBSs to enhance network coverage. As shown in Fig 1, there are two layers of BSs in the network. The first layer is the GBSs, which is modeled as 2D HPPP ${𝛷}_{G}$ on the ground with density $\lambda_{G}$ and transmission power $P_{G}$. The second layer is composed of UAVs, which are deployed on a plane with a height of *H*, and obey 2D HPPP ${𝛷}_{U}$ with density $\lambda_{U}$ and transmission power $P_{U}$. Besides, different density settings can be adopted according to different number of UAVs in actual deployment. In order to improve the network performance, the UAVs and the GBSs share the same spectrum resources that are divided into *M* channels. The orthogonal multiple access technology is used to randomly assign the available channels to users in the region. To describe the random distribution of users, their locations are modeled as a 2D HPPP ${𝛷}_{UE}$ with density $\lambda_{UE}$ on the ground. The user access to the ground network or the aerial network, and the CRE technology with bias is used to balance the load.

### Channel model

Both Large-scale and small-scale fading are considered in the channel model, where large-scale fading is determined by path loss, and small-scale fading is affected by multipath [24]. Besides, LoS transmission and Non-LoS (NLoS) transmission have different propagation characteristics according to different parameter Settings. If the transmit power is $P_{t}$, the receive power in the case of LoS and NLoS is


$$\begin{array}{c} P_{r}=\left\{ \begin{array}{c} \eta_{L}P_{t}l^{-\alpha_{L}}g_{L}\;\;\;\;\;LoS\\ \eta_{N}P_{t}l^{-\alpha_{N}}g_{N}\;\;NLoS \end{array}\right., \end{array}$$ (1)

where *l* is the distance between the user and the service BS, $\alpha_{L}$ and $\alpha_{N}$ ($2<\alpha_{L}<\alpha_{N}$) are the path loss exponent corresponding to LoS and NLoS links, $g_{L}$ and $g_{N}$ are the small-scale channel gain. For small-scale fading, the Nakagami-m distribution and Rayleigh distribution are used to represent fading in LoS and NLoS cases, respectively. For the Nakagami-m fading, $g_{L}$ is a gamma distribution and its shape and scale parameters are $m_{L}$. For the Rayleigh fading, $g_{N}$ is a exponential distribution with unit mean. Specifically, $g_{N}$ can be expressed as the gamma distribution of $m_{N}=1$. $\eta_{L}$ and $\eta_{N}$ are the path loss truncation when the reference distance is 1 m.

$g_{m}$ plays a key role in the theoretical analysis. Its Complementary Cumulative Distribution Function (CCDF) is


$$\begin{array}{c} F\left(x\right)=1-e^{-mx}\sum_{k=0}^{m-1}\frac{{\left(mx\right)}^{k}}{k!}. \end{array}$$ (2)

The above equation can be converted to


$$\begin{array}{c} F\left(x\right)=1-\frac{{\left(-1\right)}^{m-1}m^{m}}{\left(m-1\right)!}\frac{\partial^{m-1}}{\partial m^{m-1}}\frac{e^{-mx}}{m}, \end{array}$$ (3)

where *x* appears only in the exponent section. However, when *m* is larger, the computational complexity of higher derivatives is larger, and the Alzer lemma [25] gives a strict upper bound of the CCDF as


$$\begin{array}{c} F\left(x\right)⩽\sum_{n=1}^{m}C_{n}^{m}{\left(-1\right)}^{n+1}e^{-nDx}, \end{array}$$ (4)

where $D={\left(m!\right)}^{-\frac{1}{m}}m$. This formula can be used to approximate the coverage rate in the following analysis, and the equal sign is valid when *m* = 1. It can be seen that due to the randomness of small-scale fading, it is necessary to solve the Laplace transform of the interference.

For UAV, the probability $P_{U,L}\left(r\right)$ that the user under its coverage and the communication link is LoS is [26]


$$\begin{array}{c} P_{U,L}\left(r\right)=\frac{1}{1+c\exp\left(-b\left(\varphi \left(\frac{H}{r}\right)-c\right)\right)}, \end{array}$$ (5)

where $\varphi \left(x\right)=\frac{180}{\pi }arc\tan\left(x\right)$ is the elevation angle of the link *b*. *c* is a specific constant that depends on the environment. *H* is the height at which the UAV flies. *r* is the horizontal distance between the UAV and the user. It can be seen that the flight height of UAV is proportional to the LoS probability of link. Correspondingly, the probability of the NLoS link is


$$\begin{array}{c} P_{U,N}\left(r\right)=1-P_{U,L}\left(r\right). \end{array}$$ (6)

For the GBS, the LoS probability $P_{G,L}\left(r\right)$ is [27]


$$\begin{array}{c} P_{G,L}\left(r\right)=e^{-\beta r}, \end{array}$$ (7)

where β is a constant that depends on the geometry and density of the building. *r* is the distance between the GBS and the user. Thus, the NLoS probability of the link is:


$$\begin{array}{c} P_{G,N}\left(r\right)=1-P_{G,L}\left(r\right). \end{array}$$ (8)

### User access model

Assuming that each BS (ABS or GBS) works in an open access mode. Based on the principle of maximum reference signal receiving power, each user will access to the BS that makes the reference signal receiving power maximum. According to the channel model, the UAV can bring a higher LoS probability, and it is hoped to make full use of the LoS characteristics of the UAV to provide users with better communication service. Therefore, for a typical user, the reference signal received from the GBS is based on the received power under NLoS conditions, i.e.,


$$\begin{array}{c} P_{B,G}=\frac{B_{G}\eta_{N}P_{G}}{R_{G}^{\alpha_{N}}}. \end{array}$$ (9)

In contrast, the reference signal received from the UAV in LoS is


$$\begin{array}{c} P_{B,U}=\frac{B_{U}\eta_{L}P_{U}}{{\left(R_{U}^{2}+H^{2}\right)}^{\alpha_{L}/2}}, \end{array}$$ (10)

where $B_{G}$ and $B_{U}$ are the introduced bias. As can be seen from the setting of the reference signal, the user’s access depends on the distribution of the GBSs and the UAVs.

For a 2D HPPP 𝛷 with density λ, for any point on the plane, its distance from the nearest point in 𝛷 becomes an important parameter in network access. According to the Slivnyak-Mecke theorem, we can fix any point as the origin of the coordinate. Consider a point *x*_0_ in 𝛷 that is closest to the origin, and let its distance from the origin be *r*, called the closest distance. Due to the randomness of HPPP, *r* can get any value of [0,∞), but its Cumulative Distribution Function (CDF) satisfies the following rules.


$$\begin{array}{c} F\left(r\right)=P\left(r⩽R\right)=1-P\left(r>R\right)\overset{\left(a\right)}{=}1-\exp\left(-\lambda \pi r^{2}\right), \end{array}$$ (11)

where step (a) can be obtained from the distribution properties of HPPP. P(r>R) can be understood as the probability that no point appears in a circular region of radius *r*. Take the derivative of the above formula with respect to the nearest distance *r*, and the PDF of *r* is


$$\begin{array}{c} f\left(r\right)=2\lambda \pi r\exp\left(-\lambda \pi r^{2}\right). \end{array}$$ (12)

Therefore, the nearest distance between the user and the GBS is $R_{G_{0}}$, and the nearest horizontal distance between the user and the UAV is $R_{G_{0}}$. Due to the independence of GBS distribution ${𝛷}_{G}$ and UAV distribution ${𝛷}_{U}$, $R_{G_{0}}$ and $R_{U_{0}}$ are independent of each other, and their probability density functions are as follows.


$$\begin{array}{c} f_{R_{G_{0}}}\left(r\right)=2\lambda_{G}\pi r\exp\left(-\lambda_{G}\pi r^{2}\right). \end{array}$$ (13)

$$\begin{array}{c} f_{R_{U_{0}}}\left(r\right)=2\lambda_{U}\pi r\exp\left(-\lambda_{U}\pi r^{2}\right). \end{array}$$ (14)

### SINR model

For the users covered by the GBS, the signal received from the service BS will be interfered with other GBSs and UAVs using the same channel. For energy conservation, the GBS distributes its power evenly to each channel. Then, the SINR expression of the users under the GBS is


$$\begin{array}{c} SINR_{UE,G}=\frac{P_{UE,G}}{I_{GI,G}+I_{UI,G}+N_{0}}, \end{array}$$ (15)

where $P_{UE,G}$ is the received power. $I_{GI,G}$ is the interference power from the GBSs using the same channel, and $I_{UI,G}$ is the interference power from the UAVs using the same channel. *N*_0_ is the noise power.

For the users covered by the UAVs, they are also subject to interference from GBSs and UAVs using the same channel, and their SINR is


$$\begin{array}{c} SINR_{UE,U}=\frac{P_{UE,U}}{I_{UI,U}+I_{GI,U}+N_{0}}. \end{array}$$ (16)

### Network performance indicators

For the fixed data rate, the SINR demand is a constant according to the Shannon formula, and the network coverage determines whether the signal can be received normally. For a typical threshold *T*, the network throughput is expressed as


$$\begin{array}{c} 𝒯=P\left(SINR⩾T\right)\log_{2}\left(1+T\right). \end{array}$$ (17)

This paper uses the network throughput density as the throughput indicator of the system (unit: $bps/Hz/{km}^{2}$).

Traversal rate refers to the channel can traverse all fading states, that is, the maximum rate that the system can support correctly on fading channels. The traversal rate ℛ can usually be calculated by the following formula.


$$\begin{array}{cc} ℛ= & E\left[\log_{2}\left(1+SINR\right)\right]\\ = & \int_{0}^{\infty }P\left(\log_{2}\left(1+SINR\right)⩾x\right)dx\\ = & \int_{0}^{\infty }P\left(SINR⩾2^{x}-1\right)dx\\ = & \frac{1}{\ln2}\int_{0}^{\infty }\frac{P\left(SINR⩾t\right)}{1+t}dt. \end{array}$$ (18)

This paper also use the network traversal rate density as the performance indicator of the network (unit: $bps/Hz/{km}^{2}$).

## Performance analysis

### Probability of user accessing GBS

According to formulas (9) and (10), the probability of users accessing the GBS network can be obtained as follows.


PAG=P[BGηNPGRG0αN⩾BUηLPU(RU02+H2)αL/2]=P{RU02⩾[BUηLPUBGηNPGRG0αN]2/αL−H2}=(a)P(RU0⩾KG(RG0)1/2|KG(RG0)⩾0)×P(KG(RG0)⩾0)=+P(RU02⩾KG(RG0)|KG(RG0)<0)×P(KG(RG0)<0)=(b)P(RU0⩾KG(RG0)1/2|KG(RG0)⩾0)×P(KG(RG0)⩾0)+P(KG(RG0)<0)=(c)E[exp(−λUπKB(RG0))|KG(RG0)⩾0]×P(KG(RG0)⩾0)+P(KG(RG0)<0), (19)

where


$$\begin{array}{c} K_{G}\left(r\right)={\left(\frac{B_{U}\eta_{L}P_{U}}{B_{G}\eta_{N}P_{G}}r^{\alpha_{N}}\right)}^{2/\alpha_{L}}-H^{2}. \end{array}$$ (20)

Step (a) is obtained by discussing the value conditions of $K_{G}\left(R_{G_{0}}\right)$, and step (b) is obtained by setting $P\left(R_{U_{0}}^{2}⩾0\right)=1$. Step (c) is obtained according to the CCDF of $R_{U_{0}}$.


$$\begin{array}{cc} P\left(K_{G}\left(R_{G_{0}}\right)⩾0\right)= & P\left({\left(\frac{B_{U}\eta_{L}P_{U}}{B_{G}\eta_{N}P_{G}}R_{G_{0}}^{\alpha_{N}}\right)}^{2/\alpha_{L}}-H^{2}⩾0\right)\\ = & P\left(R_{G_{0}}^{2}⩾{\left(\frac{B_{G}\eta_{N}P_{G}}{B_{U}\eta_{L}P_{U}}H^{\alpha_{L}}\right)}^{2/\alpha_{N}}\right)\\ = & P\left(R_{G_{0}}⩾K_{G_{0}}^{1/2}\right)\\ \overset{\left(a\right)}{=} & \exp\left(-\lambda_{G}\pi K_{G_{0}}\right). \end{array}$$ (21)

In (21), step (a) is obtained according to the CCDF of $R_{G_{0}}$, where $K_{G_{0}}={\left(\frac{B_{G}\eta_{N}P_{G}}{B_{U}\eta_{L}P_{U}}H^{\alpha_{L}}\right)}^{2/\alpha_{N}}$. It can be further obtained that when $K_{G}\left(R_{G_{0}}\right)⩾0$, the PDF of $R_{G_{0}}$ is


$$\begin{array}{cc} f_{R_{G_{0}}}^{\prime }\left(r\right)= & \frac{2\lambda_{G}\pi r\exp\left(-\lambda_{G}\pi r^{2}\right)}{\exp\left(-\lambda_{G}\pi K_{G_{0}}\right)}\\ = & 2\lambda_{G}\pi r\exp\left(\lambda_{G}\pi K_{G_{0}}-\lambda_{G}\pi r^{2}\right). \end{array}$$ (22)

The probability of accessing the GBS can be obtained as follows.


PAG=E[exp(−πλUKG(RG0))|K(RG0)⩾0]×P(KG(RG0)⩾0)+P(KG(RG0)<0)=2πλGexp(−λGπKG0)∫KG01/2+∞r exp(πλGKG0=−πλGr2−πλUKG(r))dr+1−exp(−λGπKG0)=2πλG∫KG01/2+∞r exp(−πλGr2−πλUKG(r))dr+1−exp(−λGπKG0). (23)

When accessing the GBS, the distance distribution from the nearest GBS is


FAG(r)=P[RG0⩽r|BGηNPGRG0αN⩾BUηLPU(RU02+H2)αL/2]=(a)P[RG0⩽r,BGηNPGRG0αN⩾BUηLPU(RU02+H2)αL/2]P[BGηNPGRG0αN⩾BUηLPU(RU02+H2)αL/2]=(b)P[RG0⩽r,BGηNPGRG0αN⩾BUηLPU(RU02+H2)αL/2]PAG(r)=(c)1PAG[P(RG0⩽r,RU0⩾KG(RG0)1/2,=KG(RG0)⩾0)+P(RG0⩽r,KG(RG0)<0)]=(c)1PAG[P(RG0⩽r,RU0⩾KG(RG0)1/2,=KG(RG0)⩾0)+P(RG0⩽r,KG(RG0)<0)], (24)

where step (a) is obtained according to conditional probability, step (b) is obtained by substituting probability $P_{AG}$, and step (c) is obtained according to equation (19).

When $r<K_{G_{0}}^{1/2}$,


$$\begin{array}{cc} & P\left(R_{G_{0}}⩽r,K_{G}\left(R_{G_{0}}\right)⩾0\right)\\ = & P\left(K_{G_{0}}^{1/2}⩽R_{G_{0}}⩽r\right)=0. \end{array}$$ (25)

At this time,


$$\begin{array}{cc} F_{AG}\left(r\right)= & P\left(R_{G_{0}}⩽r,K_{G}\left(R_{G_{0}}\right)<0\right)\\ = & \frac{1}{P_{AG}}P\left(R_{G_{0}}⩽r,R_{G_{0}}<K_{G_{0}}^{1/2}\right)\\ = & \frac{1}{P_{AG}}P\left(R_{G_{0}}⩽r\right)\\ \overset{\left(a\right)}{=} & \frac{1}{P_{AG}}\left(1-\exp\left(-\lambda_{G}\pi r^{2}\right)\right), \end{array}$$ (26)

where step (a) is obtained according to the distribution of $R_{G_{0}}$.

When $r⩾K_{G_{0}}^{1/2}$,


$$\begin{array}{cc} & P\left(R_{G_{0}}⩽r,K_{G}\left(R_{G_{0}}\right)<0\right)\\ = & P\left(R_{G_{0}}⩽r,R_{G_{0}}<K_{G_{0}}^{1/2}\right)\\ = & P\left(R_{G_{0}}<K_{G_{0}}^{1/2}\right)\\ = & 1-\exp\left(-\lambda_{G}\pi K_{G_{0}}\right). \end{array}$$ (27)

=P(RG0⩽r,RU0⩾KG(RG0)1/2,KG(RG0)⩾0)=P(RU0⩾KG(RG0)1/2,KG01/2⩽RG0⩽r)=EKG01/2⩽RG0⩽r[exp(−πλUKG(RG0))]=(a)2πλG∫KG01/2rrexp(−πλGr2−πλUKG(r))dr, (28)

where step (a) is obtained according to the distribution of $R_{G_{0}}$.

From the above discussion, it can be concluded that when a user accesses the GBS, the distance distribution between the user and the GBS is as follows.


$$\begin{array}{c} f_{AG}\left(r\right)=\left\{ \begin{array}{cc} & 2\pi \lambda_{G}r\exp\left(-\pi \lambda_{G}r^{2}\right)/P_{AG}\\ r & <K_{G_{0}}^{1/2}\\ & 2\pi \lambda_{G}r\exp\left[-\pi \lambda_{G}r^{2}-\pi \lambda_{U}K\left(r\right)\right]/P_{AG}\\ r & ⩾K_{G_{0}}^{1/2} \end{array}\right. \end{array}$$ (29)

Since the GBS and the UAV share the same spectrum resources, it is necessary to consider the interference caused by the UAV. When the distance between a local UAV and its serving BS is $r_{G}$, the distance distribution between the user and the nearest UAV is


FUI(r,rG)=P[BGηNPGRG0αNRU0⩽r|RG0=rG,BGηNPGRG0αN⩾BUηLPU(RU02+H2)αL/2]=(a)P[RU0⩽r,RG0=rG,BGηNPGRG0αN⩾BUηLPU(RU02+H2)αL/2]P[RG0=rG,BGηNPGRG0αN⩾BUηLPU(RU02+H2)αL/2]=P[RU0⩽r,BGηNPGrGαN⩾BUηLPU(RU02+H2)αL/2]P[BGηNPGrGαN⩾BUηLPU(RU02+H2)αL/2], (30)

where step (a) is obtained according to conditional probability.

According to formula (19), it can be obtained


=P[BGηNPGrGαN⩾BUηLPU(RU02+H2)αL/2]=P(RU0⩾KG(rG)1/2,KG(rG)⩾0)+P(KG(rG)<0). (31)

=P[RU0⩽r,BGηNPGrGαN⩾BUηLPU(RU02+H2)αL/2]=P(RU0⩽r,RU0⩾KG(rG)1/2,KG(rG)⩾0)+P(RU0⩽r,KG(rG)<0). (32)

When $r_{G}<K_{G_{0}}^{1/2}$, $K_{G}\left(r_{G}\right)<0$, then


$$\begin{array}{c} P\left(R_{U_{0}}⩾K_{G}{\left(r_{G}\right)}^{1/2},K_{G}\left(r_{G}\right)⩾0\right)=0. \end{array}$$ (33)

$$\begin{array}{c} P\left(K_{G}\left(r_{G}\right)<0\right)=1. \end{array}$$ (34)

$$\begin{array}{c} P\left(R_{U_{0}}⩽r,R_{U_{0}}⩾K_{G}{\left(r_{G}\right)}^{1/2},K_{G}\left(r_{G}\right)⩾0\right)=0. \end{array}$$ (35)

$$\begin{array}{cc} & P\left(R_{U_{0}}⩽r,K_{G}\left(r_{G}\right)<0\right)\\ = & P\left(R_{U_{0}}⩽r\right)\\ \overset{\left(a\right)}{=} & 1-\exp\left(-\lambda_{U}\pi r^{2}\right), \end{array}$$ (36)

where step (a) is obtained by the distribution function of $R_{U_{0}}$. Thus, we can get


$$\begin{array}{c} F_{UI}\left(r,r_{G}\right)=1-\exp\left(-\lambda_{U}\pi r^{2}\right). \end{array}$$ (37)

When $r_{G}⩾K_{G_{0}}^{1/2}$, $K_{G}\left(r_{G}\right)⩾0$, then


$$\begin{array}{cc} & P\left(R_{U_{0}}⩾K_{G}{\left(r_{G}\right)}^{1/2},K_{G}\left(r_{G}\right)⩾0\right)\\ = & P\left(R_{U_{0}}⩾K_{G}{\left(r_{G}\right)}^{1/2}\right)\\ \overset{\left(a\right)}{=} & \exp\left(-\pi \lambda_{U}K_{G}\left(r_{G}\right)\right). \end{array}$$ (38)

Among them, step (a) is calculated according to the distribution function of $R_{U_{0}}$.


$$\begin{array}{c} P\left(K\left(r_{G}\right)<0\right)=0. \end{array}$$ (39)

$$\begin{array}{cc} & P\left(R_{U_{0}}⩽r,R_{U_{0}}⩾K_{G}{\left(r_{G}\right)}^{1/2},K_{G}\left(r_{G}\right)⩾0\right)\\ = & P\left(K_{G}{\left(r_{G}\right)}^{1/2}⩽R_{U_{0}}⩽r\right)\\ \overset{\left(a\right)}{=} & \exp\left(-\pi \lambda_{U}K_{G}\left(r_{G}\right)\right)-\exp\left(-\pi \lambda_{U}r^{2}\right), \end{array}$$ (40)

where, step (a) is calculated according to the distribution function of $R_{U_{0}}$.


$$\begin{array}{c} P\left(R_{U_{0}}⩽r,K_{G}\left(r_{G}\right)<0\right)=0. \end{array}$$ (41)

Thus, we can obtain


$$\begin{array}{c} F_{UI}\left(r,r_{G}\right)=\frac{\exp\left(-\pi \lambda_{U}K_{G}\left(r_{G}\right)\right)-\exp\left(-\pi \lambda_{U}r^{2}\right)}{\exp\left(-\pi \lambda_{U}K_{G}\left(r_{G}\right)\right)}. \end{array}$$ (42)

Further, the PDF of the distance between the GBS user and the UAV can be obtained as follows.


$$\begin{array}{c} f_{UI}\left(r,r_{G}\right)=\left\{ \begin{array}{c} 2\pi \lambda_{U}r\;\exp\left(-\pi \lambda_{U}r^{2}\right)\\ \;\;r\in \left[0,+\infty \right),r_{G}<K_{G_{0}}^{1/2}\\ 2\pi \lambda_{U}r\;\exp\left(\pi \lambda_{U}K_{G}\left(r_{G}\right)-\pi \lambda_{U}r^{2}\right)\\ \;\;r\in \left[K_{G}{\left(r_{G}\right)}^{1/2},+\infty \right),r_{G}⩾K_{G_{0}}^{1/2} \end{array}\right. \end{array}$$ (43)

### Probability of user accessing UAV

The probability of the user accessing the UAV network is


$$\begin{array}{cc} P_{AU}= & P\left[\frac{B_{U}\eta_{L}P_{U}}{{\left(R_{U_{0}}^{2}+H^{2}\right)}^{\alpha_{L}/2}}⩾\frac{B_{G}\eta_{N}P_{G}}{R_{G_{0}}^{\alpha_{N}}}\right]\\ = & P\left\{R_{G_{0}}⩾{\left[\frac{B_{G}\eta_{N}P_{G}}{B_{U}\eta_{L}P_{U}}{\left(R_{U_{0}}^{2}+H^{2}\right)}^{\alpha_{L}/2}\right]}^{1/\alpha_{N}}\right\}\\ \overset{\left(a\right)}{=} & E\left[\exp\left(-\pi \lambda_{G}K_{U}\left(R_{U_{0}}\right)\right)\right]\\ \overset{\left(b\right)}{=} & 2\pi \lambda_{U}\int_{0}^{+\infty }r\exp\left(-\pi \lambda_{U}r^{2}-\pi \lambda_{G}K_{U}\left(r\right)\right)dr, \end{array}$$ (44)

where step (a) is obtained by the distribution function of $R_{G_{0}}$, and step (b) is obtained by the distribution function of $R_{U_{0}}$. Among them,


$$\begin{array}{c} K_{U}\left(r\right)={\left[\frac{B_{G}\eta_{N}P_{G}}{B_{U}\eta_{L}P_{U}}{\left(r^{2}+H^{2}\right)}^{\alpha_{L}/2}\right]}^{2/\alpha_{N}}. \end{array}$$ (45)

When a UAV is connected, its horizontal distance from the nearest UAV is distributed as.


$$\begin{array}{cc} & F_{AU}\left(r\right)\\ = & P\left[R_{U_{0}}⩽r|\frac{B_{U}\eta_{L}P_{U}}{{\left(R_{U_{0}}^{2}+H^{2}\right)}^{\alpha_{L}/2}}⩾\frac{B_{G}\eta_{N}P_{G}}{R_{G_{0}}^{\alpha_{N}}}\right]\\ = & \frac{P\left[R_{U_{0}}⩽r,\frac{B_{U}\eta_{L}P_{U}}{{\left(R_{U_{0}}^{2}+H^{2}\right)}^{\alpha_{L}/2}}⩾\frac{B_{G}\eta_{N}P_{G}}{R_{G_{0}}^{\alpha_{N}}}\right]}{P\left[\frac{B_{U}\eta_{L}P_{U}}{{\left(R_{U_{0}}^{2}+H^{2}\right)}^{\alpha_{L}/2}}⩾\frac{B_{G}\eta_{N}P_{G}}{R_{G_{0}}^{\alpha_{N}}}\right]}\\ \overset{\left(b\right)}{=} & \frac{P\left[R_{U_{0}}⩽r,\frac{B_{U}\eta_{L}P_{U}}{{\left(R_{U_{0}}^{2}+H^{2}\right)}^{\alpha_{L}/2}}⩾\frac{B_{G}\eta_{N}P_{G}}{R_{G_{0}}^{\alpha_{N}}}\right]}{P_{AU}}\\ \overset{\left(c\right)}{=} & \frac{1}{P_{AU}}P\left(R_{U_{0}}⩽r,R_{G_{0}}⩾K_{U}{\left(R_{U_{0}}\right)}^{1/2}\right)\\ = & \frac{1}{P_{AU}}E_{R_{U_{0}}⩽r}\left[\exp\left(-\pi \lambda_{G}K_{U}\left(R_{U_{0}}\right)\right)\right]\\ \overset{\left(d\right)}{=} & \frac{2\pi \lambda_{U}}{P_{AU}}\int_{0}^{r}r\exp\left(-\pi \lambda_{U}r^{2}-\pi \lambda_{G}K_{U}\left(r\right)\right)dr, \end{array}$$ (46)

where step (a) is obtained according to conditional probability, step (b) is obtained by substituting probability $P_{AU}$, step (c) is obtained according to equation (44), step (d) is obtained according to the distribution of $R_{U_{0}}$. Further, it can be obtained that when the user accesses the UAV, the PDF of the horizontal distance between the user and the UAV is


$$\begin{array}{c} f_{AG}\left(r\right)=\frac{2\pi \lambda_{U}}{P_{AU}}r\exp\left[-\pi \lambda_{U}r^{2}-\pi \lambda_{G}K_{U}\left(r\right)\right]. \end{array}$$ (47)

When the horizontal distance between a user and its serving UAV is fixed, the distance distribution between it and the nearest GBS is as follows.


FGI(r,rU)=P[BGηNPGRG0αNRG0⩽r|RU0=rU,BUηLPU(RU02+H2)αL/2⩾BGηNPGRG0αN]=(a)P[RG0⩽r,RU0=rU,BUηLPU(RU02+H2)αL/2⩾BGηNPGRG0αN]P[RU0=rU,BUηLPU(RU02+H2)αL/2⩾BGηNPGRG0αN]=P[RG0⩽r,BUηLPU(rU2+H2)αL/2⩾BGηNPGRG0αN]P[BUηLPU(rU2+H2)αL/2⩾BGηNPGRG0αN], (48)

where step (a) is obtained according to conditional probability.

According to equation (44), it can be obtained


$$\begin{array}{cc} & P\left[\frac{B_{U}\eta_{L}P_{U}}{{\left(r_{U}^{2}+H^{2}\right)}^{\alpha_{L}/2}}⩾\frac{B_{G}\eta_{N}P_{G}}{R_{G_{0}}^{\alpha_{N}}}\right]\\ = & P\left(R_{G_{0}}⩾K_{U}{\left(r_{U}\right)}^{1/2}\right)\\ \overset{\left(a\right)}{=} & \exp\left(-\pi \lambda_{G}K\left(r_{U}\right)\right), \end{array}$$ (49)

where step (a) is calculated according to the distribution function of $R_{G_{0}}$.


$$\begin{array}{cc} & P\left[R_{G_{0}}⩽r,\frac{B_{U}\eta_{L}P_{U}}{{\left(r_{U}^{2}+H^{2}\right)}^{\alpha_{L}/2}}⩾\frac{B_{G}\eta_{N}P_{G}}{R_{G_{0}}^{\alpha_{N}}}\right]\\ = & P\left(R_{G_{0}}⩽r,R_{G_{0}}⩾K_{U}{\left(r_{U}\right)}^{1/2}\right)\\ \overset{\left(a\right)}{=} & \exp\left(-\pi \lambda_{G}K\left(r_{U}\right)\right)-\exp\left(-\pi \lambda_{G}r^{2}\right), \end{array}$$ (50)

where step (a) is calculated according to the distribution function of $R_{G_{0}}$. Thus, we can obtain


$$\begin{array}{c} F_{GI}\left(r,r_{U}\right)=\frac{\exp\left(-\pi \lambda_{G}K\left(r_{U}\right)\right)-\exp\left(-\pi \lambda_{G}r^{2}\right)}{\exp\left(-\pi \lambda_{G}K\left(r_{U}\right)\right)}. \end{array}$$ (51)

Furthermore, the PDF of the horizontal distance between the user and the GBS can be obtained as follows.


$$\begin{array}{c} f_{GI}\left(r,r_{U}\right)=2\pi \lambda_{G}r\exp\left(\pi \lambda_{G}K_{U}\left(r_{U}\right)-\pi \lambda_{G}r^{2}\right). \end{array}$$ (52)

### Interference BS density

For a GBS, when the number of users in its cell is $N_{UE,G}$, the probability of using a certain channel is


$$\begin{array}{cc} P_{GI,M}\left(N_{UE,G}\right)= & \frac{C_{M-1}^{\min\left(M,N_{UE,G}\right)-1}}{C_{M}^{\min\left(M,N_{UE,G}\right)}}\\ = & \frac{\min\left(M,N_{UE,G}\right)}{M}. \end{array}$$ (53)

For a UAV, when the number of users in its cell is $N_{UE,U}$, the probability of using a certain channel is


$$\begin{array}{c} P_{UI,M}\left(N_{UE,U}\right)=\frac{\min\left(M,N_{UE,U}\right)}{M}. \end{array}$$ (54)

Because the shape of the cell covered by the GBS is no longer regular, and the distribution of users is random, the number of users in the cell is a random variable. Thus, the PDF of the cell area of the GBS is


$$\begin{array}{c} f_{GS}\left(s\right)=\lambda_{G}^{3.5}\frac{3.5^{3.5}}{𝛤\left(3.5\right)}s^{2.5}\exp\left(-3.5\lambda_{G}s\right). \end{array}$$ (55)

Formula (23) deduces the probability $P_{AG}$ of users accessing the GBS. Diluted by the point process, the users covered by the GBS are equivalent to HPPP ${𝛷}_{UE,G}$ with density $\lambda_{UE,G}=P_{AG}\lambda_{UE}$. According to the distribution property of HPPP, the distribution of the number of users in any GBS cell is as follows.


$$\begin{array}{cc} & P\left(N_{UE,G}=i\right)\\ = & \int_{0}^{+\infty }\frac{{\left(\lambda_{UE,G}s\right)}^{i}}{i!}\exp\left(-\lambda_{2}s\right)f_{GS}\left(s\right)ds\\ = & \lambda_{G}^{3.5}\frac{3.5^{3.5}}{𝛤\left(3.5\right)}\int_{0}^{+\infty }\frac{{\left(\lambda_{UE,G}s\right)}^{i}}{i!}\exp\left(-\lambda_{UE,G}s\right)\\ & \times s^{2.5}\exp\left(-3.5\lambda_{G}s\right)ds\\ = & \lambda_{G}^{3.5}\frac{3.5^{3.5}\lambda_{UE,G}^{i}}{i!𝛤\left(3.5\right)}\int_{0}^{+\infty }\exp\left[-\left(3.5\lambda_{G}+\lambda_{UE,G}\right)s\right]\\ & \times s^{i+2.5}ds\\ \overset{\left(a\right)}{=} & \lambda_{G}^{3.5}\frac{3.5^{3.5}\lambda_{UE,G}^{i}}{i!𝛤\left(3.5\right){\left(3.5\lambda_{G}+\lambda_{UE,G}\right)}^{i+3.5}}\\ & \times \int_{0}^{+\infty }\exp\left(-t\right)t^{i+2.5}dt\\ = & \lambda_{G}^{3.5}\frac{3.5^{3.5}\lambda_{UE,G}^{i}}{i!{\left(3.5\lambda_{G}+\lambda_{UE,G}\right)}^{i+3.5}}\frac{𝛤\left(i+3.5\right)}{𝛤\left(3.5\right)}\\ = & \frac{{\left(3.5\lambda_{G}/\lambda_{UE,G}\right)}^{3.5}}{{\left(3.5\lambda_{G}/\lambda_{UE,G}+1\right)}^{i+3.5}i!}\frac{𝛤\left(3.5+i\right)}{𝛤\left(3.5\right)}, \end{array}$$ (56)

where step (a) is obtained by using the substitution $\left(3.5\lambda_{G}+\lambda_{UE,G}\right)s=t$. Since the channel allocation of each BS is independent, the probability of any BS using a certain channel, that is, the probability of interfering with other users in this channel, is


$$\begin{array}{cc} P_{GI}= & E\left[P_{GI,M}\left(N_{G}\right)\right]\\ = & \sum_{i=0}^{\infty }\frac{\min\left(M,i\right)}{M}P\left(N_{UE,G}=i\right)\\ = & 1-\sum_{i=0}^{M}\frac{M-i}{M}P\left(N_{UE,G}=i\right). \end{array}$$ (57)

Thus, according to the dilution calculation of the point process, the density of the interference GBS is $\lambda_{GI}=P_{GI}\lambda_{G}$.

Correspondingly, the PDF of the UAV covering the area can be obtained as follows.


$$\begin{array}{c} f_{US}\left(s\right)=\lambda_{U}^{3.5}\frac{3.5^{3.5}}{𝛤\left(3.5\right)}s^{2.5}\exp\left(-3.5\lambda_{U}s\right). \end{array}$$ (58)

where the user is equivalent to HPPP ${𝛷}_{UE,U}$ with density $\lambda_{UE,U}=P_{AU}\lambda_{UE}$. The distribution of the number of users served by UAV is as follows.


$$\begin{array}{cc} & P\left(N_{UE,U}=i\right)\\ = & \frac{{\left(3.5\lambda_{U}/\lambda_{UE,U}\right)}^{3.5}}{{\left(3.5\lambda_{U}/\lambda_{UE,U}+1\right)}^{i+3.5}i!}\frac{𝛤\left(3.5+i\right)}{𝛤\left(3.5\right)}. \end{array}$$ (59)

Since the channel assignment of each UAV is independent, the probability of any UAV using a certain channel, that is, the probability of causing interference to other users of this channel, is


$$\begin{array}{c} P_{UI}=1-\sum_{i=0}^{M}\frac{M-i}{M}P\left(N_{UE,U}=i\right). \end{array}$$ (60)

According to the dilution calculation of the point process, the density of the interference UAV is $\lambda_{UI}=P_{UI}\lambda_{U}$.

### SINR of GBS users

For GBS users, the received signal will be interfered with other GBS and UAVs using the same channel. For energy conservation, the BS distributes power evenly to each channel. The SINR expression of the GBS user can be expressed as


$$\begin{array}{c} SINR_{UE,G}=\frac{P_{UE,G}}{I_{GI,G}+I_{UI,G}+N_{0}}, \end{array}$$ (61)

where $P_{UE,G}$ is the expected received signal power, $I_{GI,G}$ is the interference signal power caused by the GBSs, $I_{UI,G}$ is the interference signal power caused by the UAV, and *N*_0_ is the noise power. For the sake of discussion, it is considered that all the interference links are NLoS.

When the distance between the user and the serving BS is $r_{G}$, its distribution area is marked as ${𝔹}_{GI,G}$, as the serving GBS is the closest, and the interference GBSs are distributed outside the circle with the radius of $r_{G}$. Meanwhile, the interference BSs obey HPPP ${𝛷}_{GI,G}$ with density $\lambda_{GI,G}=P_{GI}\lambda_{G}$ in this area. The Laplace transform of $I_{GI,G}$ is


ℒGI,G(s,rG)=E[e−sIGI,G(rG)]=E[exp(−s∑𝛷GI,GηNPGgNrαN)]=E[∏𝛷GI,Gexp(−sηNPGgNrαN)]=(a)exp{−2πλGI,G∫𝔹GI,Gr{ηNPGgNrαN1−E[exp(−sηNPGgNrαN)]}dr}=(b)exp{−2πλGI,G∫𝔹GI,Gr[ηNPGgNrαN1−LgL(sηNPGrαN)]dr}=exp{−2πλGI,G∫rG+∞r[(mNmN+sηNPGrαN)ηNPGgNrαN1−(mNmN+sηNPGrαN)mN]dr}, (62)

where step (a) is based on the Laplace function of HPPP and step (b) is based on the Laplace transform of Nakagami-m fading.

According to Formula (47), when the distance from the serving BS is $r_{G}$, the distribution of the nearest horizontal distance between the user and the UAV can be obtained. For a certain value $r_{UI}$, the interference UAV is distributed outside the circle area where *H* is the center and $r_{UI}$ is the radius. Since each UAV is assigned with an independent channel, the probability $P_{UI}$ of the UAV using the same channel as the GBS can be obtained by equation (60). It can be obtained that the distribution of the interference UAV follows HPPP ${𝛷}_{GI,G}$ with density $\lambda_{UI,G}=P_{UI}\lambda_{U}$. The Laplace transform of $I_{UI,G}$ is


ℒUI,G(s,rG)=E[e−sIUI,G(rG)]=E[exp(−s∑𝛷UI,GηNPUgN(r2+H2)αN/2)]=(a)E[exp{−2πλUI,G∫rUI+∞r[1[1−(mNmN+sηNPU(r2+H2)αN/2)mN]−(mNmN+sηNPU(r2+H2)αN/2)mN]dr}]=(b)∫0+∞fUI(t,rG)exp{[1−(mNmN+sηNPU(r2+H2)αN/2)mN]−2πλUI,G×=∫t+∞r[1−(mNmN+sηNPU(r2+H2)αN/2)mN]dr}dt, (63)

Where step (a) is obtained according to the same analysis process as formula (62), and step (b) is obtained by the horizontal distance distribution $f_{UI}\left(r,r_{G}\right)$. It can be obtained that when the distance between the user and its serving BS is $r_{G}$, the distribution of SINR is


=P(SINRUE,G⩾γ|rG)=P(PUE,G(rG)IGI,G(rG)+IUI,G(rG)+N0⩾γ)=(a)PG,L(rG)P(ηLPGrG−αLgLIGI,G(rG)+IUI,G(rG)+N0⩾γ)==+PG,NL(rG)P(ηNPGrG−αNgNIGI,G(rG)+IUI,G(rG)+N0⩾γ)=PG,L(rG)×P(gL⩾γrGαLηLPG(IGI,G(rG)+IUI,G(rG)+N0))==+PG,NL(rG)×P(gN⩾γrGαNηNPG(IGI,G(rG)+IUI,G(rG)+N0)), (64)

wehe step (a) is to separately solve whether the received signal is LoS or not. In the case of LoS, we can get


=P(gL⩾γrGαLηLPG(IGI,G(rG)+IUI,G(rG)+N0))=      =(a)∑n=1mLCnmL(−1)n+1exp(−nDmLγrGαLηLPG(IGI,G(rG)      +IUI,G(rG)+N0)nDmLγrGαLηLPG)      =(b)∑n=1mLCnmL(−1)n+1exp(−nDmLγrGαLηLPGN0)   =×ℒGI,G(nDmLγrGαLηLPG,rG)ℒUI,G(nDmLγrGαLηLPG,rG), (65)

where step (a) uses the approximation formula for the CCDF of the gamma distribution, $D_{m}={\left(m!\right)}^{-\frac{1}{m}}m$, step (b) is defined according to the Laplace transform.

Similarly, in the case of NLoS, we can get


=P(gN⩾γrGαNηNPG(IGI,G(rG)+IUI,G(rG)+N0))==∑n=1mNCnmN(−1)n+1exp(−nDmNγrGαNηLPGN0)=×ℒGI,G(nDmNγrGαNηLPG,rG)ℒUI,G(nDmNγrGαNηLPG,rG). (66)

According to the above, we can get


=P(SINRUE,G⩾γ|rG)=exp(−βrG)∑n=1mLCnmL(−1)n+1exp(−nDmLγrGαLηLPGN0)=×ℒGI,G(nDmLγrGαLηLPG,rG)ℒUI,G(nDmLγrGαLηLPG,rG)==+(1−exp(−βrG))×∑n=1mNCnmN(−1)n+1×exp(−nDmNγrGαNηLPGN0)ℒGI,G(nDmNγrGαNηLPG,rG)×ℒUI,G(nDmNγrGαNηLPG,rG). (67)

Furthermore, the SINR distribution for any user can be obtained as:


$$\begin{array}{cc} & P\left(SINR_{UE,G}⩾\gamma \right)\\ = & \int_{0}^{\infty }f_{AG}\left(r\right)P\left(SINR_{UE,G}\left(r\right)⩾\gamma |r\right)dr. \end{array}$$ (68)

As a comparison, when the network does not use UAV for auxiliary coverage, the SINR of the user covered by the GBS is:


$$\begin{array}{c} SINR_{UE,NU}=\frac{P_{UE,NU}}{I_{GI,NU}+N_{0}}. \end{array}$$ (69)

Since all users will access the GBS at this time, the PDF of the distance between any user and the nearest GBS is


$$\begin{array}{c} f_{NU}\left(r\right)=2\lambda_{G}\pi r\exp\left(-\lambda_{G}\pi r^{2}\right). \end{array}$$ (70)

For any GBS cell, the distribution of the number of users $N_{UE,NU}$ is


$$\begin{array}{c} P\left(N_{UE,NU}=i\right)=\frac{{\left(3.5\lambda_{G}/\lambda_{UE}\right)}^{3.5}}{{\left(3.5\lambda_{G}/\lambda_{UE}+1\right)}^{i+3.5}i!}\frac{𝛤\left(3.5+i\right)}{𝛤\left(3.5\right)}. \end{array}$$ (71)

The probability of any BS using a certain channel is


$$\begin{array}{c} P_{GI,NU}=1-\sum_{i=0}^{M}\frac{M-i}{M}P\left(N_{UE,NU}=i\right) \end{array}$$ (72)

HPPP ${𝛷}_{GI,NU}$ with a BS density of $\lambda_{GI,NU}=P_{GI,NU}\lambda_{G}$ is interfered. Thus, when the distance between the user and its serving BS is $r_{NU}$, the Laplace transform of interference $I_{GI,NU}$ is


$$\begin{array}{cc} & {ℒ}_{GI,NU}\left(s,r_{NU}\right)\\ = & \exp\left\{-2\pi \lambda_{GI,NU}\int_{r_{NU}}^{+\infty }r\left[1-{\left(\frac{m_{N}}{m_{N}+\frac{s\eta_{N}P_{G}}{r^{\alpha_{N}}}}\right)}^{m_{N}}\right]dr\right\}. \end{array}$$ (73)

The distribution of the SINR is


=P(SINRUE,NU⩾γ|rNU)=exp(−βrNU)∑n=1mLCnmL(−1)n+1=×exp(−nDmLγrNUαLηLPGN0)ℒGI,NU(nDmLγrNUαLηLPG,rNU)==+(1−exp(−βrNU))×∑n=1mNCnmN(−1)n+1×exp(−nDmNγrNUαNηLPGN0)ℒGI,NU(nDmNγrNUαNηLPG,rNU). (74)

Further, the SINR distribution for any user can be obtained as


$$\begin{array}{cc} & P\left(SINR_{UE,NU}⩾\gamma \right)\\ = & \int_{0}^{\infty }f_{NU}\left(r\right)P\left(SINR_{UE,NU}\left(r\right)⩾\gamma |r\right)dr. \end{array}$$ (75)

### SINR of UAV users

For UAV users, they are also subject to the interference from GBSs and UAVs using the same channel, and their SINR is


$$\begin{array}{c} SINR_{UE,U}=\frac{P_{UE,U}}{I_{UI,U}+I_{GI,U}+N_{0}}. \end{array}$$ (76)

When the horizontal distance between user and UAV is $r_{U}$, its distribution area is recorded as ${𝔹}_{UI,U}$. UAVs obey HPPP ${𝛷}_{UI,U}$ with density $\lambda_{UI,U}=P_{UI}\lambda_{U}$ in this region. Considering that the interference link is NLoS, we can adopt the same analysis process as equation (62) and get the Laplace transform of $I_{UI,U}$ as follows.


=ℒUI,U(s,rU)=E[e−sIUI,U(rU)]=E[exp(−s∑𝛷UI,UηNPUgN(r2+H2)αN/2)]=exp{−2πλUI,U∫rU+∞r[(mNmN+sηNPU(r2+H2)αN/2)1−(mNmN+sηNPU(r2+H2)αN/2)mN]dr}. (77)

Following the same analysis procedure as in equation (63), the Laplace transform of $I_{GI,U}$ can be obtained as


ℒGI,U(s,rU)=E[e−sIGI,U(rU)]=E[exp(−s∑𝛷GI,UηNPGgNrαN)]=E[exp{−2πλGI,U∫rGI+∞r[(mNmN+sηNPU(r2+H2)αN/2)1−(mNmN+sηNPGrαN)mN]dr}]=∫0+∞fGI(t,rU)exp{(mNmN+sηNPGrαN)−2πλGI,U×∫t+∞r[1−(mNmN+sηNPGrαN)mN]dr}dt. (78)

When the fixed horizontal distance is $r_{U}$, the SINR distribution of the UAV users is


=P(SINRUE,U⩾γ|rU)=P(PUE,U(rU)IUI,U(rU)+IGI,U(rU)+N0⩾γ)=(a)PU,L(rU)P(ηLPU(rU2+H2)−αL/2gLIUI,U(rU)+IGI,U(rU)+N0⩾γ)==+PU,N(rU)P(ηNPU(rU2+H2)−αN/2gNIUI,U(rU)+IGI,U(rU)+N0⩾γ)=PU,L(rU)P(gL⩾γ(rU2+H2)αL/2ηLPU×(IUI,U(rU)+IGI,U(rU)+N0)γ(rU2+H2)αL/2ηLPU)+PU,N(rU)P(gN⩾γ(rU2+H2)αN/2ηNPU×(IUI,U(rU)+IGI,U(rU)+N0)γ(rU2+H2)αL/2ηLPU). (79)

where step (a) is to solve whether the received signal is LoS or NLoS.

In the case of LoS, we can derive


P(gL⩾γ(rU2+H2)αL/2ηLPU×(IUI,U(rU)+IGI,U(rU)+N0)γ(rU2+H2)αL/2ηLPU)=(a)∑n=1mLCnmL(−1)n+1exp(nDmLγ(rU2+H2)αL/2ηLPU−nDmLγ(rU2+H2)αL/2ηLPU=×(IUI,U(rU)+IGI,U(rU)+N0)nDmLγ(rU2+H2)αL/2ηLPU)=(b)∑n=1mLCnmL(−1)n+1exp(−nDmLγ(rU2+H2)αL/2ηLPUN0)×ℒUI,U(nDmLγ(rU2+H2)αL/2ηLPU,rU)×ℒGI,U(nDmLγ(rU2+H2)αL/2ηLPU,rU). (80)

where step (a) uses the approximation formula for the CCDF of the gamma distribution, $D_{m}={\left(m!\right)}^{-\frac{1}{m}}m$, step (b) is defined according to the Laplace transform.

Similarly, in the case of NLoS, we can derive


P(gN⩾γ(rU2+H2)αN/2ηNPU×(IUI,U(rU)+IGI,U(rU)+N0)γ(rU2+H2)αL/2ηLPU)=∑n=1mNCnmN(−1)n+1exp(−nDmNγ(rU2+H2)αN/2ηNPUN0)×ℒUI,U(nDmNγ(rU2+H2)αN/2ηNPU,rU)×ℒGI,U(nDmNγ(rU2+H2)αN/2ηNPU,rU). (81)

Thus, we have


=P(SINRUE,U⩾γ|rU)=11+cexp(−b(φ(HrU)−c))=∑n=1mLCnmL(−1)n+1×exp(−nDmLγ(rU2+H2)αL/2ηLPUN0)×ℒUI,U(nDmLγ(rU2+H2)αL/2ηLPU,rU)×ℒGI,U(nDmLγ(rU2+H2)αL/2ηLPU,rU)+(1−11+cexp(−b(φ(HrU)−c)))×∑n=1mNCnmN(−1)n+1exp(−nDmNγ(rU2+H2)αN/2ηNPUN0)×ℒUI,U(nDmNγ(rU2+H2)αN/2ηNPU,rU)×ℒGI,U(nDmNγ(rU2+H2)αN/2ηNPU,rU). (82)

Further, the SINR for any user can be obtained as


$$\begin{array}{cc} & P\left(SINR_{UE,U}⩾\gamma \right)\\ = & \int_{0}^{\infty }f_{AU}\left(r\right)P\left(SINR_{UE,U}\left(r\right)⩾\gamma |r\right)dr. \end{array}$$ (83)

### Network throughput and traversal rate

For GBS users, the distribution is equivalent to HPPP ${𝛷}_{UE,G}$ with density $\lambda_{UE,G}=P_{AG}\lambda_{UE}$. For a BS, the number of users it can cover is $\min\left(M,N_{UE,G}\right)$, which is expressed as


$$\begin{array}{cc} & E\left[\min\left(M,N_{UE,G}\right)\right]\\ = & M-\sum_{i=0}^{M}\left(M-i\right)P\left(N_{UE,G}=i\right). \end{array}$$ (84)

Since the density of the BS is $\lambda_{G}$, according to the SINR distribution of a single user, the throughput density under the coverage of the GBS is


$$\begin{array}{cc} {𝒯}_{G}= & \lambda_{G}E\left[\min\left(M,N_{UE,G}\right)\right]\\ & \times \log_{2}\left(1+\gamma \right)P\left(SINR_{UE,G}⩾\gamma \right). \end{array}$$ (85)

Similarly for UAV users, the distribution is equivalent to HPPP ${𝛷}_{UE,U}$ with density $\lambda_{UE,U}=P_{AU}\lambda_{UE}$. For a certain UAV, the number of users it can cover is $\min\left(M,N_{UE,U}\right)$, which is expressed as:


$$\begin{array}{cc} & E\left[\min\left(M,N_{UE,U}\right)\right]\\ = & M-\sum_{i=0}^{M}\left(M-i\right)P\left(N_{UE,U}=i\right). \end{array}$$ (86)

The throughput density under UAV coverage can be obtained as follows.


$$\begin{array}{cc} {𝒯}_{U}= & \lambda_{U}E\left[\min\left(M,N_{UE,U}\right)\right]\\ & \times \log_{2}\left(1+\gamma \right)P\left(SINR_{UE,U}⩾\gamma \right). \end{array}$$ (87)

Therefore, the total throughput density of the system is


$$\begin{array}{c} {𝒯}_{UU}={𝒯}_{G}+{𝒯}_{U}. \end{array}$$ (88)

For comparison, when the system does not use UAVs, the average number of users covered by the GBSs is


$$\begin{array}{c} E\left[\min\left(M,N_{UE,NU}\right)\right] \end{array}$$ (89)

$$\begin{array}{c} =M-\sum_{i=0}^{M}\left(M-i\right)P\left(N_{UE,NU}=i\right). \end{array}$$ (90)

Thus, the network throughput is


$$\begin{array}{cc} {𝒯}_{NU}= & \lambda_{G}E\left[\min\left(M,N_{UE,NU}\right)\right]\\ & \times \log_{2}\left(1+\gamma \right)P\left(SINR_{UE,NU}⩾\gamma \right). \end{array}$$ (91)

For ground network, the network traversal rate density is


$$\begin{array}{cc} {ℛ}_{B}= & \frac{\lambda_{U}E\left[\min\left(M,N_{UE,U}\right)\right]}{M\ln2}\\ & \times \int_{0}^{\infty }\frac{P\left(SINR_{UE,U}⩾t\right)}{1+t}dt. \end{array}$$ (92)

For the UAV network, the network traversal rate density is


$$\begin{array}{cc} {ℛ}_{U}= & \frac{\lambda_{G}E\left[\min\left(M,N_{UE,NU}\right)\right]}{M\ln2}\\ & \times \int_{0}^{\infty }\frac{P\left(SINR_{UE,NU}⩾\gamma \right)}{1+t}dt. \end{array}$$ (93)

Thus, the total traversal rate density of the UAV-assisted network can be obtained as follows.


$$\begin{array}{c} {ℛ}_{UU}={ℛ}_{G}+{ℛ}_{U}. \end{array}$$ (94)

As a comparison, for ground network without UAV, the network traversal rate density is


$$\begin{array}{cc} {ℛ}_{NU}= & \frac{\lambda_{G}E\left[\min\left(M,N_{UE,NU}\right)\right]}{M\ln2}\\ & \times \int_{0}^{\infty }\frac{P\left(SINR_{UE,NU}⩾t\right)}{1+t}dt. \end{array}$$ (95)

### Simulation results

In order to verify the correctness of the above theoretical analysis, this section uses Monte Carlo simulation. Firstly, the simulated network scene is introduced, and then the simulated network performance is obtained. After verifying the accuracy of the theoretical analysis, the influence of the network parameters such as the flight height of the UAV, the density of the UAV and the GBS, and the multiplicative bias on the coverage performance is analyzed and discussed. Unless otherwise specified, the simulation parameter settings in each result are shown in Table 1 [28].

**Table 1:** Simulation Parameters Setting.

variable	value	unit	variable	value	unit
*b*	0.136	1	$\lambda_{UE}$	10	*km* ^2^
*c*	11.95	1	$\lambda_{G}$	1	*km* ^2^
1/β	3141.4	1	$\lambda_{U}$	3	*km* ^2^
$\alpha_{L}$	2.5	1	$P_{G}$	20	W
$\alpha_{N}$	4	1	$P_{U}$	5	W
$\eta_{L}$	10^−5.11^	1	*N*_0_	−84	dBm
$\eta_{N}$	10^−10^	1	*H*	300	m
$m_{L}$	3	1	*B*	3	dB
$m_{N}$	1	1	*M*	5	1

The simulation tool is using MATLAB, and the simulation times are set to 10000 times. In the simulation process, first set the density of the user, the UAV and the GBS, the transmission power and the relative bias of the UAV and the GBS. The square area with a side length of 20000 m is taken as the simulation area, and then the area in the whole simulation is generated according to the density of the GBS, the UAV and the user according to the HPPP process. Since the user’s service BS is selected from the nearest GBS and UAV, each user first finds the GBS and UAV with the closest distance, then calculates the received signal power and reference signal strength respectively, and then chooses to access the GBS network and UAV network. Each GBS and UAV can randomly assign a channel to the user who chooses it as the service BS. In this way, the channel assignment of the whole network can be determined.

In order to better simulate the extensive interference of each user, a new square area with a center side length of 5000 m is selected as the typical area, where the user is the typical user. First of all, for the typical user of each channel, it can be known whether it is covered by GBS or UAV according to its access situation. Further, according to the received signal power and the transmission power of other BSs using the same channel, the SINR can be obtained to determine whether the SINR exceeds threshold. If so, the typical user is successfully covered. In this way, the number of users covered by GBS or UAV in a typical area, the number of users assigned to the channel, and the number of users successfully covered can be obtained.

**Fig 2:**
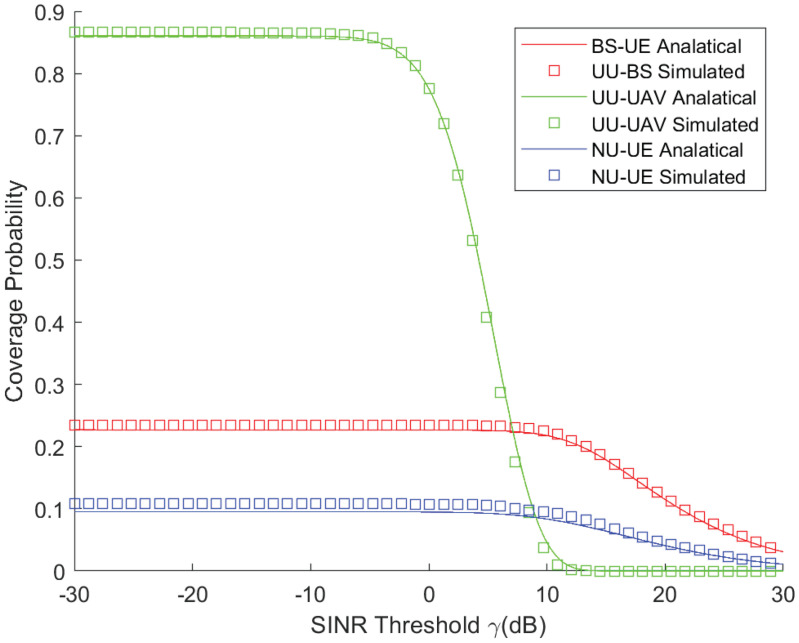
Coverage probability vs. SINR threshold.

**Fig 3:**
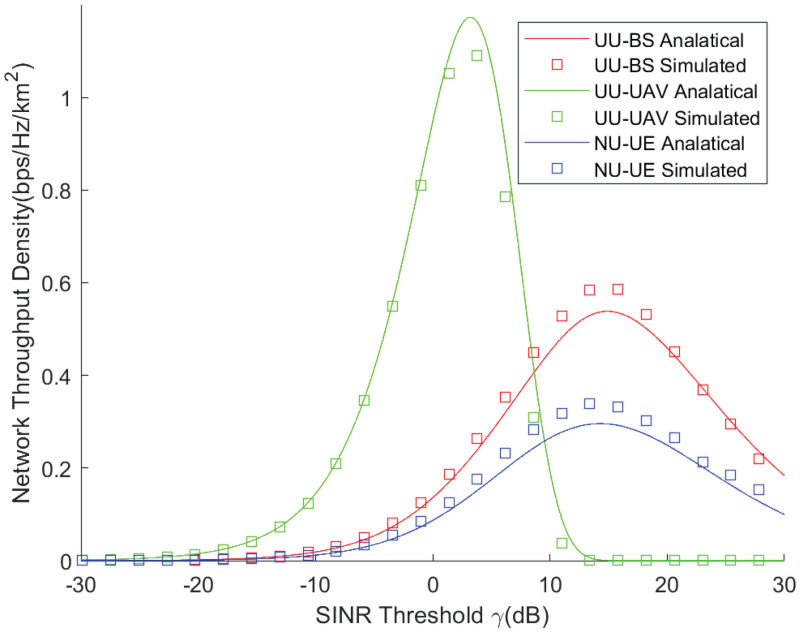
Network throughput density vs. SINR threshold.

Fig 2 and Fig 3 show the simulation and theoretical results of target user coverage and network throughput density under the proposed scheme and the comparison schemes. They indicate that the theoretical values are basically consistent with the simulation results, which confirms the correctness of the theoretical analysis. It can be seen that the UAV has higher coverage probability and network throughput density for users, and the GBS can also provide better SINR for users who choose to access.

**Fig 4:**
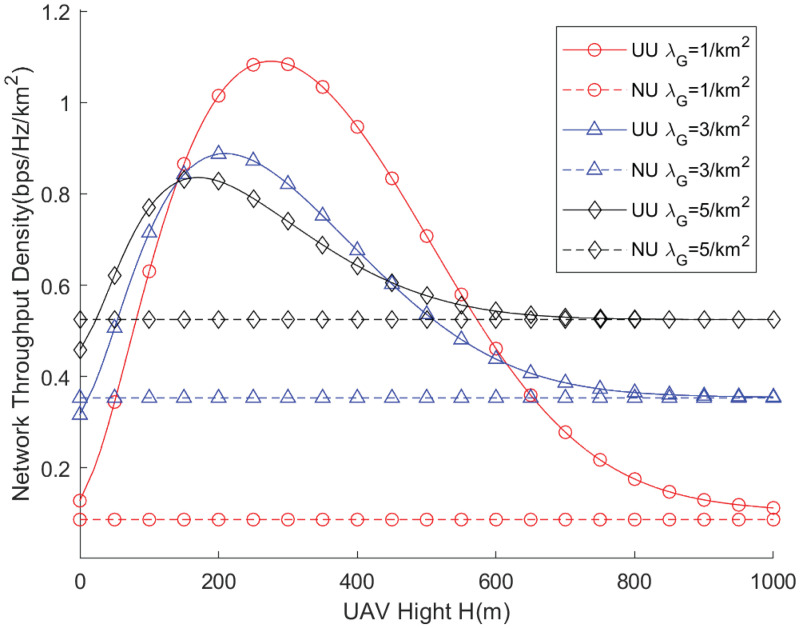
Network throughput density vs. UAV height.

**Fig 5:**
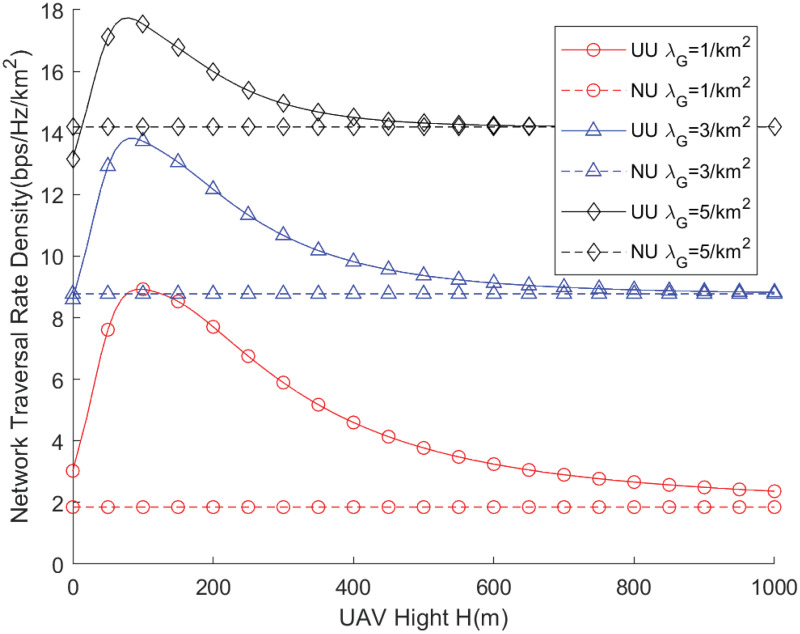
Network traversal rate density vs. UAV height.

Fig 4 and Fig 5 show the influence of UAV height on network performance under different density of GBS through theoretical calculation. As can be seen from the figure, when the density of GBSs increases, the network has better performance. In terms of network throughput, since users’ communication rate requirements are fixed, increasing the density of GBSs will make more users access to the GBS network, thus reducing the impact of UAVs on network performance. For the network traversal rate density, increasing the GBS density can effectively improve the network performance. From the impact of UAV height on performance, it can be seen that if the UAV height is not properly set in the deployment of the UAV, the network performance will not be well improved.

**Fig 6:**
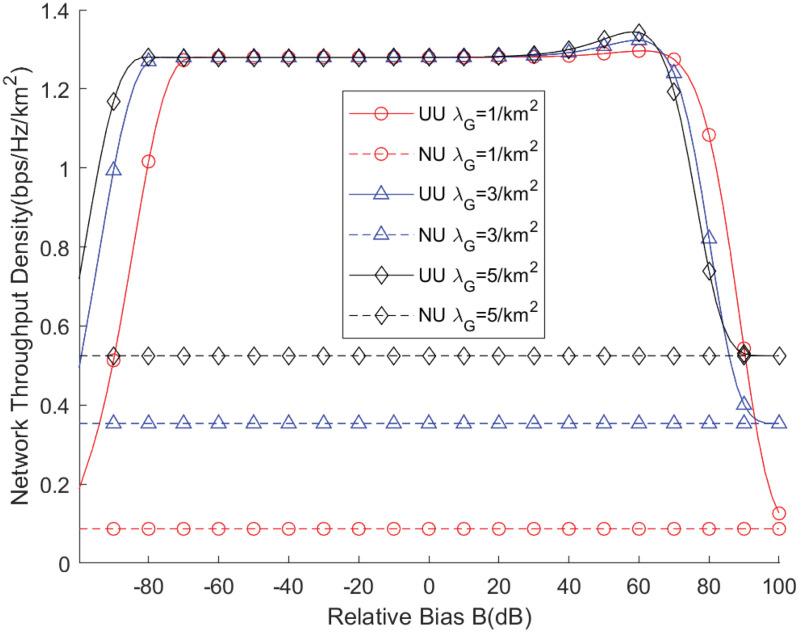
Network throughput density vs. Relative bias.

**Fig 7:**
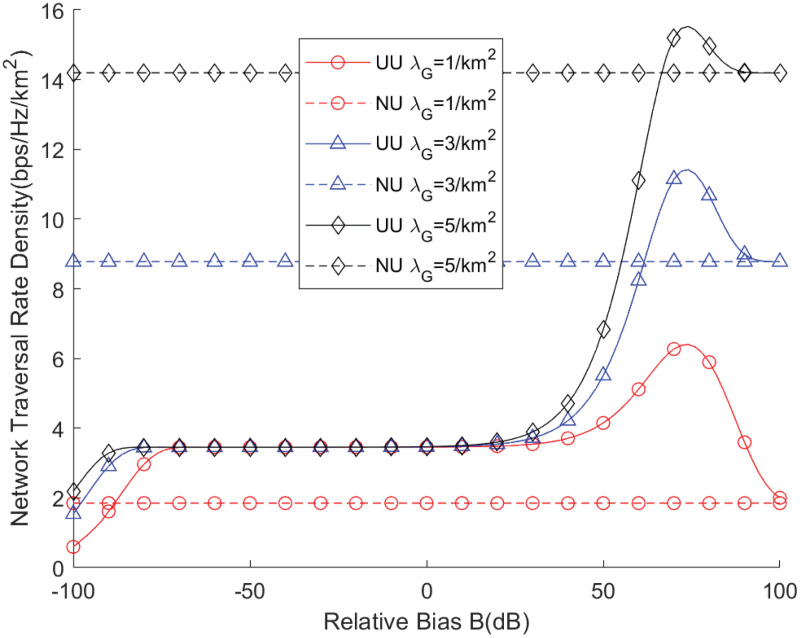
Network traversal rate density vs. Relative bias.

Fig 6 and Fig 7 show the influence of relative bias on network performance under different density of GBSs through theoretical calculation. As can be seen from the figure, in order to balance the coverage performance of UAVs and GBSs, the network has the best bias setting. At the same time, it can be seen that if the bias is not properly set, the network performance will deteriorate. This is because when the relative bias is too large, a large number of users will access to the GBS network, making the UAV network lose its function. When the relative bias is too low, a large number of users access to the UAV network, making the GBS network lose its function. Therefore, in order to make full use of air-and-ground integrated network, it is necessary to set the relative bias reasonably.

**Fig 8:**
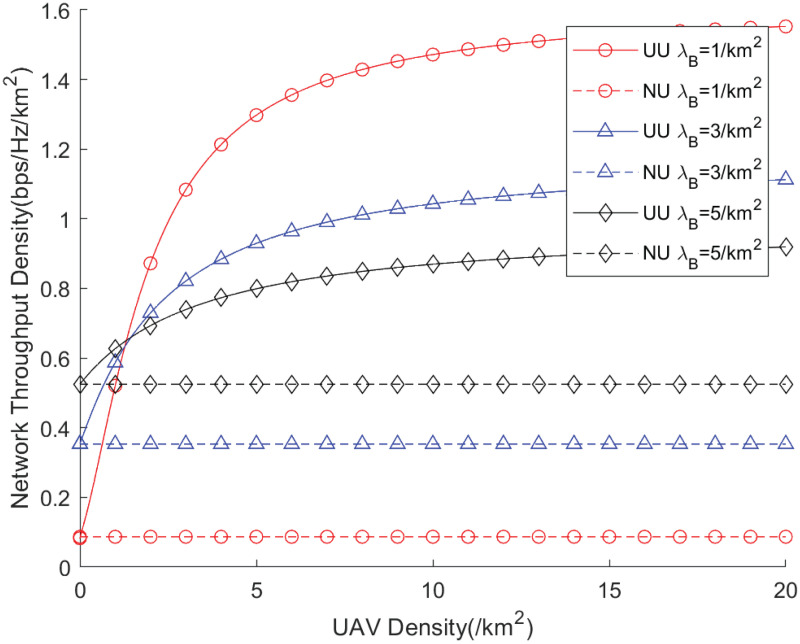
Network throughput density vs. UAV density.

**Fig 9:**
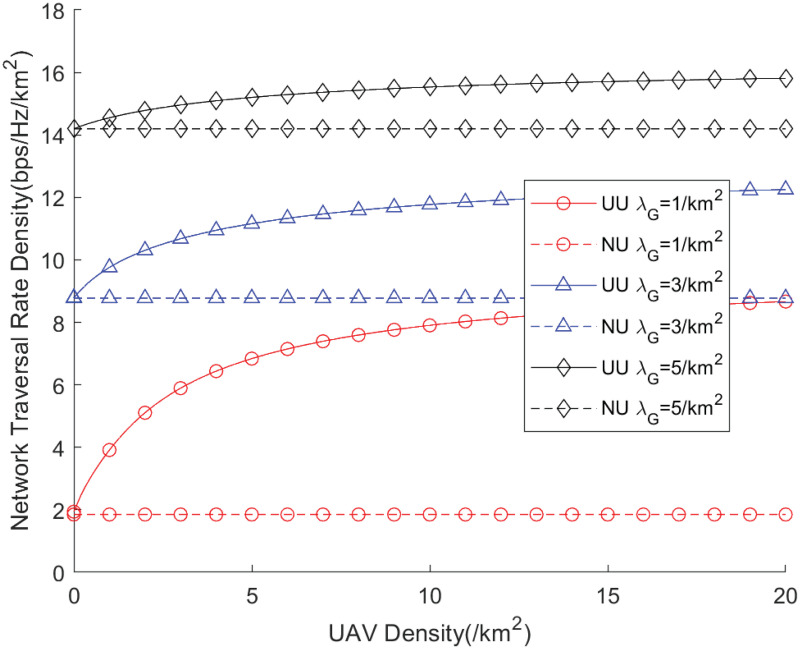
Network traversal rate density vs. UAV density.

Fig 8 and Fig 9 show the influence of UAV density on network performance under different densities of GBSs through theoretical calculation. It can be seen that the improvement of UAV density can effectively improve network performance, especially when the density of GBSs is low, the increase of UAV density can enable more users to access to the UAV link and provide better coverage performance for users. When the density of GBSs is high, the coverage performance of UAVs will be limited. In this case, the network performance should be optimized by changing other parameters, such as multiplicative bias.

**Fig 10:**
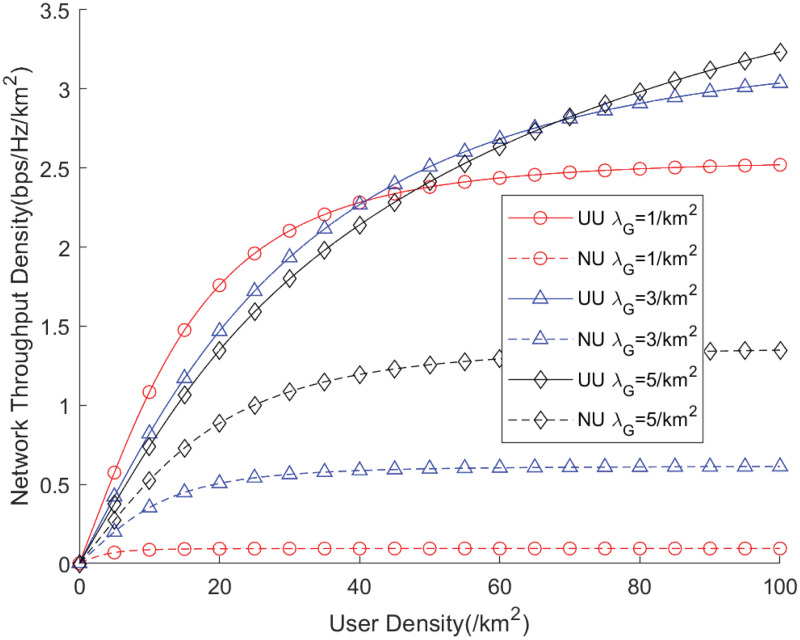
Network throughput density vs. User density.

**Fig 11:**
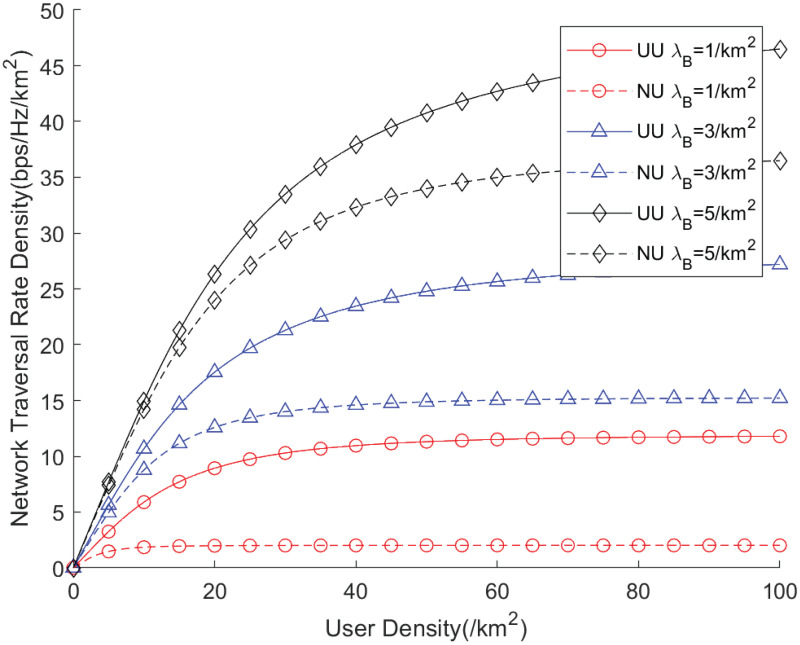
Network traversal rate density vs. User density.

Fig 10 and Fig 11 show the influence of UAV density on network performance under different densities of GBSs through theoretical calculation. As can be seen from the figure, since the UAV reuses the spectrum resources of the GBS, the network can provide coverage for more users, and the network performance has been improved exponentially. In contrast, when only GBSs are used for coverage, the network will quickly saturate with increasing user density. At the same time, in terms of network throughput density, the higher the density of GBSs, the better the performance, so the deployment of BSs should be reasonable according to the actual situation of the network in the actual deployment.

**Fig 12:**
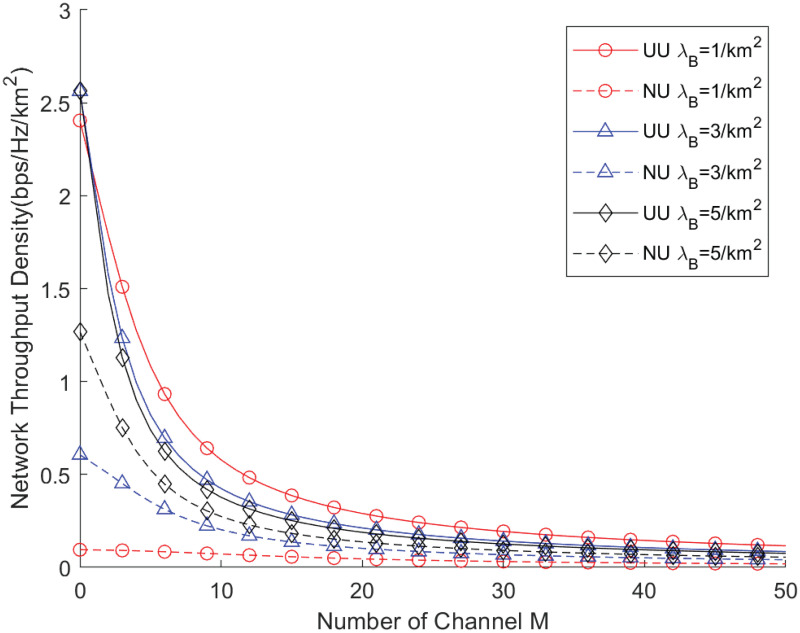
Network throughput density vs. Number of channel.

**Fig 13:**
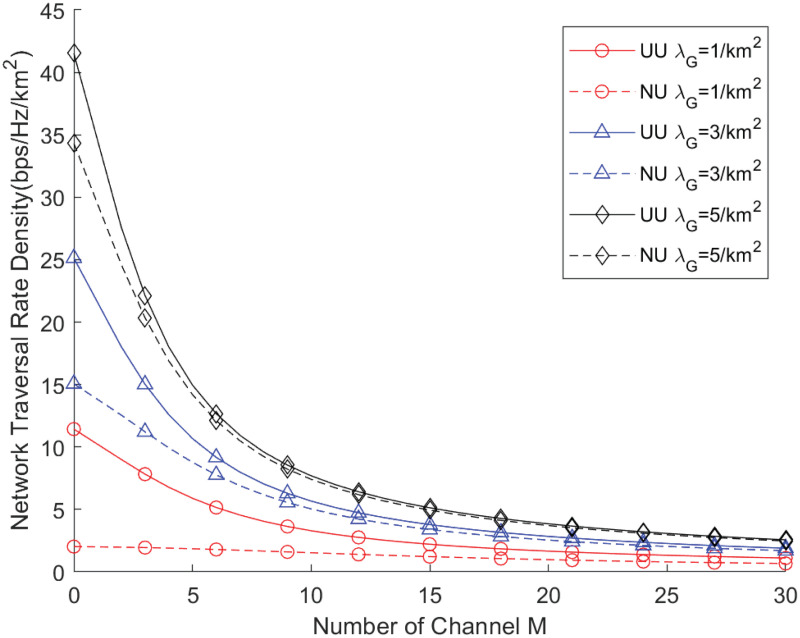
Network traversal rate density vs. Number of channel.

Fig 12 and Fig 13 show the influence of channel number on network performance under different density of GBSs through theoretical calculation. It can be seen that when the network is not saturated, increasing the number of channels will make redundant channels idle and reduce the performance of the network. Therefore, in the network setting, the number of channels needs to be allocated according to the density of users, so that the network can give full play to the performance, and also provide better coverage for more users.

## Conclusion

This paper analyzes the UAV-assisted coverage in sparse networks, and uses CRE technology to coordinate the coverage performance of the heterogeneous networks that are jointly covered by UAVs and GBSs. The stochastic geometry theory is used to analyze the throughput density and traversal rate density under each network coverage. Both theoretical analysis and simulation results show that the proposed scheme can improve the network performance. The effects of various parameters on network performance are analyzed through theoretical results. The multiplicative bias introduced in CRE technology can coordinate networks well when the network deployment changes, and jointly provide users with excellent coverage performance. At the same time, because the UAV network reuses the spectrum resources of the GBS network, the auxiliary coverage of the UAV network can bring huge traffic improvement to the sparse network.

In future work, we will take into account the uneven user distribution and irregular base station coverage when analyzing and designing integrated air-and-ground networks. Furthermore, multi-layer aerial networks constructed by combining high-altitude and low-altitude nodes will help further enhance the overall network coverage performance. Specifically, the reliability of backhaul links and the efficiency of the cooperation mechanism between high-altitude and low-altitude nodes are regarded as the decisive factors affecting system operation. In addition, the limited battery capacity leads to prominent energy consumption issues for UAV platforms, which cannot be ignored in practical deployment. Therefore, how to reasonably design and deploy aerial networks under tight energy constraints is extremely crucial for guaranteeing stable and sustainable network services.

## Supporting information

S1 File Source code for simulation results. We conduct simulations with MATLAB R2024b. The source code for Figs.2–13 are provided in the supporting file.
(PDF)
