# Supplementary material for: Modeling and analysis of UAV-Assisted sparse ground networks
Source: PLoS One. 2026 Jul 15;21(7):e0352585. doi: 10.1371/journal.pone.0352585 (PMC13372247; doi:10.1371/journal.pone.0352585)
Supplement: S1 File — We conduct simulations with MATLAB R2024b. The source code for Figs.2–13 are provided in the supporting file. (PDF) [file pone.0352585.s001.pdf]

Fig.2 Coverage probability vs. SINR threshold

```
BS_lam=1e-6;
UAV_lam=3e-6;
UE_lam=10e-6;
B=80;
B_BS=10.^(B./10);
B_UAV=1;
KK_l=10.^(3./10);
KK_nl=10.^(23./10);
m_l=3;
m_nl=1;
a_l=2.5;
a_nl=4;
K_l=1./(KK_l.*(4.*pi.*2.*10.^9./(3.*10.^8)).^a_l);
K_nl=1./(KK_nl.*(4.*pi.*2.*10.^9./(3.*10.^8)).^a_nl);
P_UAV_UE=5;
H_UAV_UE=300;
m_I=m_nl;
a_I=a_nl;
K_I=K_nl;
R_BS=100;
R_UAV=200;
R_0=300;
P_BS_UE=20;
P_BS_I=P_BS_UE;
N_cha=5;
UAV_cover_thet=pi./8;
sig2=10.^(-114./10);
gam_db=linspace(-30,30,500);
% gam_db=0;
UE_gam=10.^(gam_db./10);
% UE_gam=linspace(0,100,10)


[P_BS_a]=HPPP_two_BS_P_ana(UE_lam,UE_gam,sig2,N_cha,...

m_l,m_nl,a_l,a_nl,K_l,K_nl,...

B_BS,BS_lam,P_BS_UE,...
```

```
B_UAV,UAV_lam,P_UAV_UE,H_UAV_UE);

[P_BS_s]=HPPP_two_BS_P_simu(UE_lam,UE_gam,sig2,N_cha,..
.
m_l,m_nl,a_l,a_nl,K_l,K_nl,...
B_BS,BS_lam,P_BS_UE,...
B_UAV,UAV_lam,P_UAV_UE,H_UAV_UE);


[P_UAV_a]=HPPP_two_UAV_P_ana(UE_lam,UE_gam,sig2,N_cha,..
.
m_l,m_nl,a_l,a_nl,K_l,K_nl,...
B_BS,BS_lam,P_BS_UE,...
B_UAV,UAV_lam,P_UAV_UE,H_UAV_UE);

[P_UAV_s]=HPPP_two_UAV_P_simu(UE_lam,UE_gam,sig2,N_cha,.
..
m_l,m_nl,a_l,a_nl,K_l,K_nl,...
B_BS,BS_lam,P_BS_UE,...
B_UAV,UAV_lam,P_UAV_UE,H_UAV_UE);
[P_NU_a]=NU_HPPP_two_BS_P_ana(UE_lam,UE_gam,sig2,N_cha,.
..
m_l,m_nl,a_l,a_nl,K_l,K_nl,...
B_BS,BS_lam,P_BS_UE,...
B_UAV,UAV_lam,P_UAV_UE,H_UAV_UE);
```

```
[P_NU_s]=NU_HPPP_two_BS_P_simu(UE_lam,UE_gam,sig2,N_cha,
...

m_l,m_nl,a_l,a_nl,K_l,K_nl,...

B_BS,BS_lam,P_BS_UE,...

B_UAV,UAV_lam,P_UAV_UE,H_UAV_UE);


figure
hold
N=3;
plot(gam_db,P_BS_a,'r-');
plot(gam_db(1:N:end),P_BS_s(1:N:end),'rs');
plot(gam_db,P_UAV_a,'g-');
plot(gam_db(1:N:end),P_UAV_s(1:N:end),'gs')
plot(gam_db,P_NU_a,'b-');
plot(gam_db(1:N:end),P_NU_s(1:N:end),'bs');
```

Fig.3 Network throughput density vs. SINR threshold

```
BS_lam=1e-6;
UAV_lam=3e-6;
UE_lam=10e-6;
B=80;
B_BS=10.^(B./10);
B_UAV=1;
KK_l=10.^(3./10);
KK_nl=10.^(23./10);
m_l=3;
m_nl=1;
a_l=2.5;
a_nl=4;
K_l=1./(KK_l.*(4.*pi.*2.*10.^9./(3.*10.^8)).^a_l);
K_nl=1./(KK_nl.*(4.*pi.*2.*10.^9./(3.*10.^8)).^a_nl);
P_UAV_UE=5;
```

```
H_UAV_UE=300;
m_I=m_nl;
a_I=a_nl;
K_I=K_nl;
R_BS=100;
R_UAV=200;
R_0=300;
P_BS_UE=20;
P_BS_I=P_BS_UE;
N_cha=5;
% UAV_cover_thet=pi./8;
sig2=10.^(-114./10);
gam_db=linspace(-30,30,500);
% gam_db=0;
UE_gam=10.^(gam_db./10);
% UE_gam=linspace(0,100,10)

[T_BS_a]=HPPP_two_BS_T_ana(UE_lam,UE_gam,sig2,N_cha,...

m_l,m_nl,a_l,a_nl,K_l,K_nl,...

B_BS,BS_lam,P_BS_UE,...

B_UAV,UAV_lam,P_UAV_UE,H_UAV_UE);


[T_BS_s]=HPPP_two_BS_T_simu(UE_lam,UE_gam,sig2,N_cha,..
.

m_l,m_nl,a_l,a_nl,K_l,K_nl,...

B_BS,BS_lam,P_BS_UE,...

B_UAV,UAV_lam,P_UAV_UE,H_UAV_UE);



[T_UAV_a]=HPPP_two_UAV_T_ana(UE_lam,UE_gam,sig2,N_cha,..
.

m_l,m_nl,a_l,a_nl,K_l,K_nl,...
```

```
B_BS,BS_lam,P_BS_UE,...

B_UAV,UAV_lam,P_UAV_UE,H_UAV_UE);


[T_UAV_s]=HPPP_two_UAV_T_simu(UE_lam,UE_gam,sig2,N_cha,.
..

m_l,m_nl,a_l,a_nl,K_l,K_nl,...

B_BS,BS_lam,P_BS_UE,...

B_UAV,UAV_lam,P_UAV_UE,H_UAV_UE);


[T_NU_a]=NU_HPPP_two_BS_T_ana(UE_lam,UE_gam,sig2,N_cha,.
..

m_l,m_nl,a_l,a_nl,K_l,K_nl,...

B_BS,BS_lam,P_BS_UE,...

B_UAV,UAV_lam,P_UAV_UE,H_UAV_UE);


[T_NU_s]=NU_HPPP_two_BS_T_simu(UE_lam,UE_gam,sig2,N_cha,
...

m_l,m_nl,a_l,a_nl,K_l,K_nl,...

B_BS,BS_lam,P_BS_UE,...

B_UAV,UAV_lam,P_UAV_UE,H_UAV_UE);


figure
hold
N=8;
plot(gam_db,T_BS_a.*1e6,'r-');
plot(gam_db(1:N:end),T_BS_s(1:N:end).*1e6,'rs');
```

```
plot(gam_db,T_UAV_a.*1e6,'g-');
plot(gam_db(1:N:end),T_UAV_s(1:N:end).*1e6,'gs')
plot(gam_db,T_NU_a.*1e6,'b-');
plot(gam_db(1:N:end),T_NU_s(1:N:end).*1e6,'bs');
```

Fig.4 Network throughput density vs. UAV height

```
BS_lam=1e-6;
UAV_lam=3e-6;
UE_lam=10e-6;
B=80;
B_BS=10.^(B./10);
B_UAV=1;
KK_l=10.^(3./10);
KK_nl=10.^(23./10);
m_l=3;
m_nl=1;
a_l=2.5;
a_nl=4;
K_l=1./(KK_l.*(4.*pi.*2.*10.^9./(3.*10.^8)).^a_l);
K_nl=1./(KK_nl.*(4.*pi.*2.*10.^9./(3.*10.^8)).^a_nl);
P_UAV_UE=5;
H_UAV_UE=300;
m_I=m_nl;
a_I=a_nl;
K_I=K_nl;
P_BS_UE=20;
N_cha=5;
sig2=10.^(-114./10);
gam_db=0;
UE_gam=10.^(gam_db./10);
H_UAV_UE=0:10:1000;
N_bian=H_UAV_UE;
NN=length(N_bian);

T_BS_a_1=NaN(1,NN);

T_UAV_a_1=NaN(1,NN);
```

```
T_NU_a_1=NaN(1,NN);

T_UU_a_1=NaN(1,NN);

T_BS_a_2=NaN(1,NN);

T_UAV_a_2=NaN(1,NN);

T_NU_a_2=NaN(1,NN);

T_UU_a_2=NaN(1,NN);
T_BS_a_3=NaN(1,NN);

T_UAV_a_3=NaN(1,NN);

T_NU_a_3=NaN(1,NN);

T_UU_a_3=NaN(1,NN);


parfor i=2:NN

BS_lam=1e-6;


[T_BS_a_1(i)]=HPPP_two_BS_T_ana(UE_lam,UE_gam,sig2,N_ch
a,...

m_l,m_nl,a_l,a_nl,K_l,K_nl,...

B_BS,BS_lam,P_BS_UE,...

B_UAV,UAV_lam,P_UAV_UE,H_UAV_UE(i));


[T_UAV_a_1(i)]=HPPP_two_UAV_T_ana(UE_lam,UE_gam,sig2,N_
cha,...

m_l,m_nl,a_l,a_nl,K_l,K_nl,...

B_BS,BS_lam,P_BS_UE,...
```

```
B_UAV,UAV_lam,P_UAV_UE,H_UAV_UE(i));


[T_NU_a_1(i)]=NU_HPPP_two_BS_T_ana(UE_lam,UE_gam,sig2,N
_cha,...

m_l,m_nl,a_l,a_nl,K_l,K_nl,...

B_BS,BS_lam,P_BS_UE,...

B_UAV,UAV_lam,P_UAV_UE,H_UAV_UE(i));


T_UU_a_1(i)=T_BS_a_1(i)+T_UAV_a_1(i);


BS_lam=3e-6;


[T_BS_a_2(i)]=HPPP_two_BS_T_ana(UE_lam,UE_gam,sig2,N_ch
a,...

m_l,m_nl,a_l,a_nl,K_l,K_nl,...

B_BS,BS_lam,P_BS_UE,...

B_UAV,UAV_lam,P_UAV_UE,H_UAV_UE(i));


[T_UAV_a_2(i)]=HPPP_two_UAV_T_ana(UE_lam,UE_gam,sig2,N_
cha,...

m_l,m_nl,a_l,a_nl,K_l,K_nl,...

B_BS,BS_lam,P_BS_UE,...
```

```
B_UAV,UAV_lam,P_UAV_UE,H_UAV_UE(i));


[T_NU_a_2(i)]=NU_HPPP_two_BS_T_ana(UE_lam,UE_gam,sig2,N
_cha,...

m_l,m_nl,a_l,a_nl,K_l,K_nl,...

B_BS,BS_lam,P_BS_UE,...

B_UAV,UAV_lam,P_UAV_UE,H_UAV_UE(i));


T_UU_a_2(i)=T_BS_a_2(i)+T_UAV_a_2(i);



BS_lam=5e-6;



[T_BS_a_3(i)]=HPPP_two_BS_T_ana(UE_lam,UE_gam,sig2,N_ch
a,...

m_l,m_nl,a_l,a_nl,K_l,K_nl,...

B_BS,BS_lam,P_BS_UE,...

B_UAV,UAV_lam,P_UAV_UE,H_UAV_UE(i));


[T_UAV_a_3(i)]=HPPP_two_UAV_T_ana(UE_lam,UE_gam,sig2,N_
cha,...

m_l,m_nl,a_l,a_nl,K_l,K_nl,...

B_BS,BS_lam,P_BS_UE,...

B_UAV,UAV_lam,P_UAV_UE,H_UAV_UE(i));
```

```
[T_NU_a_3(i)]=NU_HPPP_two_BS_T_ana(UE_lam,UE_gam,sig2,N
_cha,...

m_l,m_nl,a_l,a_nl,K_l,K_nl,...

B_BS,BS_lam,P_BS_UE,...

B_UAV,UAV_lam,P_UAV_UE,H_UAV_UE(i));

T_UU_a_3(i)=T_BS_a_3(i)+T_UAV_a_3(i);

i

end

T_BS_a_1(1)=2*T_BS_a_1(2)-T_BS_a_1(3);

T_UAV_a_1(1)=2*T_UAV_a_1(2)-T_UAV_a_1(3);

T_NU_a_1(1)=2*T_NU_a_1(2)-T_NU_a_1(3);
T_UU_a_1(1)=2*T_UU_a_1(2)-T_UU_a_1(3);

T_BS_a_2(1)=2*T_BS_a_2(2)-T_BS_a_2(3);

T_UAV_a_2(1)=2*T_UAV_a_2(2)-T_UAV_a_2(3);

T_NU_a_2(1)=2*T_NU_a_2(2)-T_NU_a_2(3);
T_UU_a_2(1)=2*T_UU_a_2(2)-T_UU_a_2(3);


T_BS_a_3(1)=2*T_BS_a_3(2)-T_BS_a_3(3);

T_UAV_a_3(1)=2*T_UAV_a_3(2)-T_UAV_a_3(3);

T_NU_a_3(1)=2*T_NU_a_3(2)-T_NU_a_3(3);
T_UU_a_3(1)=2*T_UU_a_3(2)-T_UU_a_3(3);
```

```
figure
hold
N=3;
plot(N_bian,T_UU_a_1,'r-');
% plot(gam_db(1:N:end),T_D_s_1(1:N:end),'rs');
plot(N_bian,T_NU_a_1,'r--');
plot(N_bian,T_UU_a_2,'b-');
plot(N_bian,T_NU_a_2,'b--');
plot(N_bian,T_UU_a_3,'k-');
plot(N_bian,T_NU_a_3,'k--');
```

Fig.5 Network traversal rate density vs. UAV height

```
BS_lam=1e-6;
UAV_lam=3e-6;
UE_lam=10e-6;
B=80;
B_BS=10.^(B./10);
B_UAV=1;
KK_l=10.^(3./10);
KK_nl=10.^(23./10);
m_l=3;
m_nl=1;
a_l=2.5;
a_nl=4;
K_l=1./(KK_l.*(4.*pi.*2.*10.^9./(3.*10.^8)).^a_l);
K_nl=1./(KK_nl.*(4.*pi.*2.*10.^9./(3.*10.^8)).^a_nl);
P_UAV_UE=5;
H_UAV_UE=300;
m_I=m_nl;
a_I=a_nl;
K_I=K_nl;
P_BS_UE=20;
N_cha=5;
sig2=10.^(-114./10);
gam_db=0;
```

```
UE_gam=10.^(gam_db./10);


H_UAV_UE=0:10:1000;
N_bian=H_UAV_UE;
NN=length(N_bian);

C_BS_a_1=NaN(1,NN);

C_UAV_a_1=NaN(1,NN);

C_NU_a_1=NaN(1,NN);

C_UU_a_1=NaN(1,NN);

C_BS_a_2=NaN(1,NN);

C_UAV_a_2=NaN(1,NN);

C_NU_a_2=NaN(1,NN);

C_UU_a_2=NaN(1,NN);
C_BS_a_3=NaN(1,NN);

C_UAV_a_3=NaN(1,NN);

C_NU_a_3=NaN(1,NN);

C_UU_a_3=NaN(1,NN);

h=0.01;
parfor i=2:NN

BS_lam=1e-6;

[C_BS_a_1(i)]=C_HPPP_two_BS_T_ana(UE_lam,h,sig2,N_cha,..
.

m_l,m_nl,a_l,a_nl,K_l,K_nl,...

B_BS,BS_lam,P_BS_UE,...
```

```
B_UAV,UAV_lam,P_UAV_UE,H_UAV_UE(i));

[C_UAV_a_1(i)]=C_HPPP_two_UAV_T_ana(UE_lam,h,sig2,N_cha,
...
m_l,m_nl,a_l,a_nl,K_l,K_nl,...
B_BS,BS_lam,P_BS_UE,...
B_UAV,UAV_lam,P_UAV_UE,H_UAV_UE(i));


[C_NU_a_1(i)]=C_NU_HPPP_two_BS_T_ana(UE_lam,h,sig2,N_ch
a,...
m_l,m_nl,a_l,a_nl,K_l,K_nl,...
B_BS,BS_lam,P_BS_UE,...
B_UAV,UAV_lam,P_UAV_UE,H_UAV_UE(i));


C_UU_a_1(i)=C_BS_a_1(i)+C_UAV_a_1(i);


BS_lam=3e-6;


[C_BS_a_2(i)]=C_HPPP_two_BS_T_ana(UE_lam,h,sig2,N_cha,..
.
m_l,m_nl,a_l,a_nl,K_l,K_nl,...
B_BS,BS_lam,P_BS_UE,...
```

```
B_UAV,UAV_lam,P_UAV_UE,H_UAV_UE(i));

[C_UAV_a_2(i)]=C_HPPP_two_UAV_T_ana(UE_lam,h,sig2,N_cha,...
m_l,m_nl,a_l,a_nl,K_l,K_nl,...
B_BS,BS_lam,P_BS_UE,...
B_UAV,UAV_lam,P_UAV_UE,H_UAV_UE(i));

[C_NU_a_2(i)]=C_NU_HPPP_two_BS_T_ana(UE_lam,h,sig2,N_cha,...
m_l,m_nl,a_l,a_nl,K_l,K_nl,...
B_BS,BS_lam,P_BS_UE,...
B_UAV,UAV_lam,P_UAV_UE,H_UAV_UE(i));

C_UU_a_2(i)=C_BS_a_2(i)+C_UAV_a_2(i);

BS_lam=5e-6;

[C_BS_a_3(i)]=C_HPPP_two_BS_T_ana(UE_lam,h,sig2,N_cha,...
m_l,m_nl,a_l,a_nl,K_l,K_nl,...
B_BS,BS_lam,P_BS_UE,...
B_UAV,UAV_lam,P_UAV_UE,H_UAV_UE(i));
```

```
[C_UAV_a_3(i)]=C_HPPP_two_UAV_T_ana(UE_lam,h,sig2,N_cha,...

m_l,m_nl,a_l,a_nl,K_l,K_nl,...

B_BS,BS_lam,P_BS_UE,...

B_UAV,UAV_lam,P_UAV_UE,H_UAV_UE(i));


[C_NU_a_3(i)]=C_NU_HPPP_two_BS_T_ana(UE_lam,h,sig2,N_cha,...

m_l,m_nl,a_l,a_nl,K_l,K_nl,...

B_BS,BS_lam,P_BS_UE,...

B_UAV,UAV_lam,P_UAV_UE,H_UAV_UE(i));


C_UU_a_3(i)=C_BS_a_3(i)+C_UAV_a_3(i);

i

end

C_BS_a_1(1)=2*C_BS_a_1(2)-C_BS_a_1(3);

C_UAV_a_1(1)=2*C_UAV_a_1(2)-C_UAV_a_1(3);

C_NU_a_1(1)=2*C_NU_a_1(2)-C_NU_a_1(3);
C_UU_a_1(1)=2*C_UU_a_1(2)-C_UU_a_1(3);

C_BS_a_2(1)=2*C_BS_a_2(2)-C_BS_a_2(3);

C_UAV_a_2(1)=2*C_UAV_a_2(2)-C_UAV_a_2(3);
```

```
C_NU_a_2(1)=2*C_NU_a_2(2)-C_NU_a_2(3);
C_UU_a_2(1)=2*C_UU_a_2(2)-C_UU_a_2(3);


C_BS_a_3(1)=2*C_BS_a_3(2)-C_BS_a_3(3);

C_UAV_a_3(1)=2*C_UAV_a_3(2)-C_UAV_a_3(3);

C_NU_a_3(1)=2*C_NU_a_3(2)-C_NU_a_3(3);
C_UU_a_3(1)=2*C_UU_a_3(2)-C_UU_a_3(3);

figure
hold
N=3;
plot(N_bian,C_UU_a_1.*1e6,'r-');
plot(N_bian,C_NU_a_1.*1e6,'r--');
plot(N_bian,C_UU_a_2.*1e6,'b-');
plot(N_bian,C_NU_a_2.*1e6,'b--');
plot(N_bian,C_UU_a_3.*1e6,'k-');
plot(N_bian,C_NU_a_3.*1e6,'k--');
```

Fig.6 Network throughput density vs. Relative bias

```
BS_lam=1e-6;
UAV_lam=3e-6;
UE_lam=10e-6;
B=80;
B_BS=10.^(B./10);
B_UAV=1;
KK_l=10.^(3./10);
KK_nl=10.^(23./10);
m_l=3;
m_nl=1;
a_l=2.5;
a_nl=4;
K_l=1./(KK_l.*(4.*pi.*2.*10.^9./(3.*10.^8)).^a_l);
K_nl=1./(KK_nl.*(4.*pi.*2.*10.^9./(3.*10.^8)).^a_nl);
P_UAV_UE=5;
H_UAV_UE=300;
```

```
m_I=m_nl;

a_I=a_nl;

K_I=K_nl;
P_BS_UE=20;
N_cha=5;
sig2=10.^(-114./10);
gam_db=0;
UE_gam=10.^(gam_db./10);
B=-100:2:100;
B_BS=10.^(B./10);


N_bian=B;
NN=length(N_bian);

T_BS_a_1=NaN(1,NN);

T_UAV_a_1=NaN(1,NN);

T_NU_a_1=NaN(1,NN);

T_UU_a_1=NaN(1,NN);

T_BS_a_2=NaN(1,NN);

T_UAV_a_2=NaN(1,NN);

T_NU_a_2=NaN(1,NN);

T_UU_a_2=NaN(1,NN);
T_BS_a_3=NaN(1,NN);

T_UAV_a_3=NaN(1,NN);

T_NU_a_3=NaN(1,NN);

T_UU_a_3=NaN(1,NN);
```

```
parfor i=2:NN

BS_lam=1e-6;



[T_BS_a_1(i)]=HPPP_two_BS_T_ana(UE_lam,UE_gam,sig2,N_ch
a,...

m_l,m_nl,a_l,a_nl,K_l,K_nl,...

B_BS(i),BS_lam,P_BS_UE,...

B_UAV,UAV_lam,P_UAV_UE,H_UAV_UE);


[T_UAV_a_1(i)]=HPPP_two_UAV_T_ana(UE_lam,UE_gam,sig2,N_
cha,...

m_l,m_nl,a_l,a_nl,K_l,K_nl,...

B_BS(i),BS_lam,P_BS_UE,...

B_UAV,UAV_lam,P_UAV_UE,H_UAV_UE);



[T_NU_a_1(i)]=NU_HPPP_two_BS_T_ana(UE_lam,UE_gam,sig2,N
_cha,...

m_l,m_nl,a_l,a_nl,K_l,K_nl,...

B_BS(i),BS_lam,P_BS_UE,...

B_UAV,UAV_lam,P_UAV_UE,H_UAV_UE);



T_UU_a_1(i)=T_BS_a_1(i)+T_UAV_a_1(i);
```

```
BS_lam=3e-6;

[T_BS_a_2(i)]=HPPP_two_BS_T_ana(UE_lam,UE_gam,sig2,N_ch
a,...
m_l,m_nl,a_l,a_nl,K_l,K_nl,...
B_BS(i),BS_lam,P_BS_UE,...
B_UAV,UAV_lam,P_UAV_UE,H_UAV_UE);

[T_UAV_a_2(i)]=HPPP_two_UAV_T_ana(UE_lam,UE_gam,sig2,N_
cha,...
m_l,m_nl,a_l,a_nl,K_l,K_nl,...
B_BS(i),BS_lam,P_BS_UE,...
B_UAV,UAV_lam,P_UAV_UE,H_UAV_UE);

[T_NU_a_2(i)]=NU_HPPP_two_BS_T_ana(UE_lam,UE_gam,sig2,N
_cha,...
m_l,m_nl,a_l,a_nl,K_l,K_nl,...
B_BS(i),BS_lam,P_BS_UE,...
B_UAV,UAV_lam,P_UAV_UE,H_UAV_UE);

T_UU_a_2(i)=T_BS_a_2(i)+T_UAV_a_2(i);
```

```
BS_lam=5e-6;


[T_BS_a_3(i)]=HPPP_two_BS_T_ana(UE_lam,UE_gam,sig2,N_ch
a,...

m_l,m_nl,a_l,a_nl,K_l,K_nl,...

B_BS(i),BS_lam,P_BS_UE,...

B_UAV,UAV_lam,P_UAV_UE,H_UAV_UE);


[T_UAV_a_3(i)]=HPPP_two_UAV_T_ana(UE_lam,UE_gam,sig2,N_
cha,...

m_l,m_nl,a_l,a_nl,K_l,K_nl,...

B_BS(i),BS_lam,P_BS_UE,...

B_UAV,UAV_lam,P_UAV_UE,H_UAV_UE);



[T_NU_a_3(i)]=NU_HPPP_two_BS_T_ana(UE_lam,UE_gam,sig2,N
_cha,...

m_l,m_nl,a_l,a_nl,K_l,K_nl,...

B_BS(i),BS_lam,P_BS_UE,...

B_UAV,UAV_lam,P_UAV_UE,H_UAV_UE);



T_UU_a_3(i)=T_BS_a_3(i)+T_UAV_a_3(i);


I
```

```
end

T_BS_a_1(1)=2*T_BS_a_1(2)-T_BS_a_1(3);

T_UAV_a_1(1)=2*T_UAV_a_1(2)-T_UAV_a_1(3);

T_NU_a_1(1)=2*T_NU_a_1(2)-T_NU_a_1(3);
T_UU_a_1(1)=2*T_UU_a_1(2)-T_UU_a_1(3);

T_BS_a_2(1)=2*T_BS_a_2(2)-T_BS_a_2(3);

T_UAV_a_2(1)=2*T_UAV_a_2(2)-T_UAV_a_2(3);

T_NU_a_2(1)=2*T_NU_a_2(2)-T_NU_a_2(3);
T_UU_a_2(1)=2*T_UU_a_2(2)-T_UU_a_2(3);


T_BS_a_3(1)=2*T_BS_a_3(2)-T_BS_a_3(3);

T_UAV_a_3(1)=2*T_UAV_a_3(2)-T_UAV_a_3(3);

T_NU_a_3(1)=2*T_NU_a_3(2)-T_NU_a_3(3);
T_UU_a_3(1)=2*T_UU_a_3(2)-T_UU_a_3(3);

figure
hold
N=3;
plot(N_bian,T_UU_a_1.*1e6,'r-');
plot(N_bian,T_NU_a_1.*1e6,'r--');
plot(N_bian,T_UU_a_2.*1e6,'b-');
plot(N_bian,T_NU_a_2.*1e6,'b--');
plot(N_bian,T_UU_a_3.*1e6,'k-');
plot(N_bian,T_NU_a_3.*1e6,'k--');
```

Fig.7 Network traversal rate density vs. Relative bias

```
BS_lam=1e-6;
UAV_lam=3e-6;
```

```
UE_lam=10e-6;
B=80;
B_BS=10.^(B./10);
B_UAV=1;
KK_l=10.^(3./10);
KK_nl=10.^(23./10);
m_l=3;
m_nl=1;
a_l=2.5;
a_nl=4;
K_l=1./(KK_l.*(4.*pi.*2.*10.^9./(3.*10.^8)).^a_l);
K_nl=1./(KK_nl.*(4.*pi.*2.*10.^9./(3.*10.^8)).^a_nl);
P_UAV_UE=5;
H_UAV_UE=300;
m_I=m_nl;
a_I=a_nl;
K_I=K_nl;
P_BS_UE=20;
N_cha=5;
sig2=10.^(-114./10);
gam_db=0;
UE_gam=10.^(gam_db./10);
B=-100:2:100;
B_BS=10.^(B./10);

N_bian=B;
NN=length(N_bian);

C_BS_a_1=NaN(1,NN);

C_UAV_a_1=NaN(1,NN);

C_NU_a_1=NaN(1,NN);

C_UU_a_1=NaN(1,NN);

C_BS_a_2=NaN(1,NN);

C_UAV_a_2=NaN(1,NN);

C_NU_a_2=NaN(1,NN);
```

```
C_UU_a_2=NaN(1,NN);
C_BS_a_3=NaN(1,NN);

C_UAV_a_3=NaN(1,NN);

C_NU_a_3=NaN(1,NN);

C_UU_a_3=NaN(1,NN);

h=0.01;
parfor i=2:NN

BS_lam=1e-6;



[C_BS_a_1(i)]=C_HPPP_two_BS_T_ana(UE_lam,h,sig2,N_cha,..
.

m_l,m_nl,a_l,a_nl,K_l,K_nl,...

B_BS(i),BS_lam,P_BS_UE,...

B_UAV,UAV_lam,P_UAV_UE,H_UAV_UE);


[C_UAV_a_1(i)]=C_HPPP_two_UAV_T_ana(UE_lam,h,sig2,N_cha,
...

m_l,m_nl,a_l,a_nl,K_l,K_nl,...

B_BS(i),BS_lam,P_BS_UE,...

B_UAV,UAV_lam,P_UAV_UE,H_UAV_UE);


[C_NU_a_1(i)]=C_NU_HPPP_two_BS_T_ana(UE_lam,h,sig2,N_ch
a,...
```

```
m_l,m_nl,a_l,a_nl,K_l,K_nl,...
B_BS(i),BS_lam,P_BS_UE,...
B_UAV,UAV_lam,P_UAV_UE,H_UAV_UE);

C_UU_a_1(i)=C_BS_a_1(i)+C_UAV_a_1(i);

BS_lam=3e-6;

[C_BS_a_2(i)]=C_HPPP_two_BS_T_ana(UE_lam,h,sig2,N_cha,..
.
m_l,m_nl,a_l,a_nl,K_l,K_nl,...
B_BS(i),BS_lam,P_BS_UE,...
B_UAV,UAV_lam,P_UAV_UE,H_UAV_UE);

[C_UAV_a_2(i)]=C_HPPP_two_UAV_T_ana(UE_lam,h,sig2,N_cha,
...
m_l,m_nl,a_l,a_nl,K_l,K_nl,...
B_BS(i),BS_lam,P_BS_UE,...
B_UAV,UAV_lam,P_UAV_UE,H_UAV_UE);

[C_NU_a_2(i)]=C_NU_HPPP_two_BS_T_ana(UE_lam,h,sig2,N_ch
a,...
```

```
m_l,m_nl,a_l,a_nl,K_l,K_nl,...
B_BS(i),BS_lam,P_BS_UE,...
B_UAV,UAV_lam,P_UAV_UE,H_UAV_UE);

C_UU_a_2(i)=C_BS_a_2(i)+C_UAV_a_2(i);


BS_lam=5e-6;


[C_BS_a_3(i)]=C_HPPP_two_BS_T_ana(UE_lam,h,sig2,N_cha,..
.
m_l,m_nl,a_l,a_nl,K_l,K_nl,...
B_BS(i),BS_lam,P_BS_UE,...
B_UAV,UAV_lam,P_UAV_UE,H_UAV_UE);

[C_UAV_a_3(i)]=C_HPPP_two_UAV_T_ana(UE_lam,h,sig2,N_cha,
...
m_l,m_nl,a_l,a_nl,K_l,K_nl,...
B_BS(i),BS_lam,P_BS_UE,...
B_UAV,UAV_lam,P_UAV_UE,H_UAV_UE);


[C_NU_a_3(i)]=C_NU_HPPP_two_BS_T_ana(UE_lam,h,sig2,N_ch
a,...
m_l,m_nl,a_l,a_nl,K_l,K_nl,...
```

```
B_BS(i),BS_lam,P_BS_UE,...

B_UAV,UAV_lam,P_UAV_UE,H_UAV_UE);

C_UU_a_3(i)=C_BS_a_3(i)+C_UAV_a_3(i);

i

end

C_BS_a_1(1)=2*C_BS_a_1(2)-C_BS_a_1(3);

C_UAV_a_1(1)=2*C_UAV_a_1(2)-C_UAV_a_1(3);

C_NU_a_1(1)=2*C_NU_a_1(2)-C_NU_a_1(3);
C_UU_a_1(1)=2*C_UU_a_1(2)-C_UU_a_1(3);

C_BS_a_2(1)=2*C_BS_a_2(2)-C_BS_a_2(3);

C_UAV_a_2(1)=2*C_UAV_a_2(2)-C_UAV_a_2(3);

C_NU_a_2(1)=2*C_NU_a_2(2)-C_NU_a_2(3);
C_UU_a_2(1)=2*C_UU_a_2(2)-C_UU_a_2(3);


C_BS_a_3(1)=2*C_BS_a_3(2)-C_BS_a_3(3);

C_UAV_a_3(1)=2*C_UAV_a_3(2)-C_UAV_a_3(3);

C_NU_a_3(1)=2*C_NU_a_3(2)-C_NU_a_3(3);
C_UU_a_3(1)=2*C_UU_a_3(2)-C_UU_a_3(3);

figure
hold
N=3;
plot(N_bian,C_UU_a_1.*1e6,'r-');
plot(N_bian,C_NU_a_1.*1e6,'r--');
plot(N_bian,C_UU_a_2.*1e6,'b-');
plot(N_bian,C_NU_a_2.*1e6,'b--');
```

```
plot(N_bian,C_UU_a_3.*1e6,'k-');
plot(N_bian,C_NU_a_3.*1e6,'k--');
```

Fig.8 Network throughput density vs. UAV density

```
BS_lam=1e-6;
UAV_lam=3e-6;
UE_lam=10e-6;
B=80;
B_BS=10.^(B./10);
B_UAV=1;
KK_l=10.^(3./10);
KK_nl=10.^(23./10);
m_l=3;
m_nl=1;
a_l=2.5;
a_nl=4;
K_l=1./(KK_l.*(4.*pi.*2.*10.^9./(3.*10.^8)).^a_l);
K_nl=1./(KK_nl.*(4.*pi.*2.*10.^9./(3.*10.^8)).^a_nl);
P_UAV_UE=5;
H_UAV_UE=300;
m_I=m_nl;
a_I=a_nl;
K_I=K_nl;
P_BS_UE=20;
N_cha=5;
sig2=10.^(-114./10);
gam_db=0;
UE_gam=10.^(gam_db./10);
UAV_lam=(0:0.1:10).*1e-6;

N_bian=UAV_lam.*1e6;
NN=length(N_bian);

T_BS_a_1=NaN(1,NN);

T_UAV_a_1=NaN(1,NN);
```

```
T_NU_a_1=NaN(1,NN);

T_UU_a_1=NaN(1,NN);

T_BS_a_2=NaN(1,NN);

T_UAV_a_2=NaN(1,NN);

T_NU_a_2=NaN(1,NN);

T_UU_a_2=NaN(1,NN);
T_BS_a_3=NaN(1,NN);

T_UAV_a_3=NaN(1,NN);

T_NU_a_3=NaN(1,NN);

T_UU_a_3=NaN(1,NN);


parfor i=2:NN

BS_lam=1e-6;

[T_BS_a_1(i)]=HPPP_two_BS_T_ana(UE_lam,UE_gam,sig2,N_ch
a,...

m_l,m_nl,a_l,a_nl,K_l,K_nl,...

B_BS,BS_lam,P_BS_UE,...

B_UAV,UAV_lam(i),P_UAV_UE,H_UAV_UE);


[T_UAV_a_1(i)]=HPPP_two_UAV_T_ana(UE_lam,UE_gam,sig2,N_
cha,...

m_l,m_nl,a_l,a_nl,K_l,K_nl,...

B_BS,BS_lam,P_BS_UE,...
```

```
B_UAV,UAV_lam(i),P_UAV_UE,H_UAV_UE);

[T_NU_a_1(i)]=NU_HPPP_two_BS_T_ana(UE_lam,UE_gam,sig2,N
_cha,...
m_l,m_nl,a_l,a_nl,K_l,K_nl,...
B_BS,BS_lam,P_BS_UE,...
B_UAV,UAV_lam(i),P_UAV_UE,H_UAV_UE);

T_UU_a_1(i)=T_BS_a_1(i)+T_UAV_a_1(i);

BS_lam=3e-6;

[T_BS_a_2(i)]=HPPP_two_BS_T_ana(UE_lam,UE_gam,sig2,N_ch
a,...
m_l,m_nl,a_l,a_nl,K_l,K_nl,...
B_BS,BS_lam,P_BS_UE,...
B_UAV,UAV_lam(i),P_UAV_UE,H_UAV_UE);

[T_UAV_a_2(i)]=HPPP_two_UAV_T_ana(UE_lam,UE_gam,sig2,N_
cha,...
m_l,m_nl,a_l,a_nl,K_l,K_nl,...
B_BS,BS_lam,P_BS_UE,...
```

```
B_UAV,UAV_lam(i),P_UAV_UE,H_UAV_UE);

[T_NU_a_2(i)]=NU_HPPP_two_BS_T_ana(UE_lam,UE_gam,sig2,N
_cha,...
m_l,m_nl,a_l,a_nl,K_l,K_nl,...
B_BS,BS_lam,P_BS_UE,...
B_UAV,UAV_lam(i),P_UAV_UE,H_UAV_UE);

T_UU_a_2(i)=T_BS_a_2(i)+T_UAV_a_2(i);

BS_lam=5e-6;

[T_BS_a_3(i)]=HPPP_two_BS_T_ana(UE_lam,UE_gam,sig2,N_ch
a,...
m_l,m_nl,a_l,a_nl,K_l,K_nl,...
B_BS,BS_lam,P_BS_UE,...
B_UAV,UAV_lam(i),P_UAV_UE,H_UAV_UE);

[T_UAV_a_3(i)]=HPPP_two_UAV_T_ana(UE_lam,UE_gam,sig2,N_
cha,...
m_l,m_nl,a_l,a_nl,K_l,K_nl,...
B_BS,BS_lam,P_BS_UE,...
B_UAV,UAV_lam(i),P_UAV_UE,H_UAV_UE);
```

```
[T_NU_a_3(i)]=NU_HPPP_two_BS_T_ana(UE_lam,UE_gam,sig2,N
_cha,...

m_l,m_nl,a_l,a_nl,K_l,K_nl,...

B_BS,BS_lam,P_BS_UE,...

B_UAV,UAV_lam(i),P_UAV_UE,H_UAV_UE);

T_UU_a_3(i)=T_BS_a_3(i)+T_UAV_a_3(i);

i
end

T_BS_a_1(1)=2*T_BS_a_1(2)-T_BS_a_1(3);

T_UAV_a_1(1)=2*T_UAV_a_1(2)-T_UAV_a_1(3);

T_NU_a_1(1)=2*T_NU_a_1(2)-T_NU_a_1(3);
T_UU_a_1(1)=2*T_UU_a_1(2)-T_UU_a_1(3);

T_BS_a_2(1)=2*T_BS_a_2(2)-T_BS_a_2(3);

T_UAV_a_2(1)=2*T_UAV_a_2(2)-T_UAV_a_2(3);

T_NU_a_2(1)=2*T_NU_a_2(2)-T_NU_a_2(3);
T_UU_a_2(1)=2*T_UU_a_2(2)-T_UU_a_2(3);


T_BS_a_3(1)=2*T_BS_a_3(2)-T_BS_a_3(3);

T_UAV_a_3(1)=2*T_UAV_a_3(2)-T_UAV_a_3(3);

T_NU_a_3(1)=2*T_NU_a_3(2)-T_NU_a_3(3);
T_UU_a_3(1)=2*T_UU_a_3(2)-T_UU_a_3(3);
```

```
figure
hold
N=3;
plot(N_bian,T_UU_a_1.*1e6,'r-');
plot(N_bian,T_NU_a_1.*1e6,'r--');
plot(N_bian,T_UU_a_2.*1e6,'b-');
plot(N_bian,T_NU_a_2.*1e6,'b--');

plot(N_bian,T_UU_a_3.*1e6,'k-');
plot(N_bian,T_NU_a_3.*1e6,'k--');
```

Fig.9 Network traversal rate density vs. UAV density

```
BS_lam=1e-6;
UAV_lam=3e-6;
UE_lam=10e-6;
B=80;
B_BS=10.^(B./10);
B_UAV=1;
KK_l=10.^(3./10);
KK_nl=10.^(23./10);
m_l=3;
m_nl=1;
a_l=2.5;
a_nl=4;
K_l=1./(KK_l.*(4.*pi.*2.*10.^9./(3.*10.^8)).^a_l);
K_nl=1./(KK_nl.*(4.*pi.*2.*10.^9./(3.*10.^8)).^a_nl);
P_UAV_UE=5;
H_UAV_UE=300;
m_I=m_nl;
a_I=a_nl;
K_I=K_nl;
P_BS_UE=20;
N_cha=5;
sig2=10.^(-114./10);
gam_db=0;
UE_gam=10.^(gam_db./10);
```

```
UAV_lam=(0:0.1:10).*1e-6;



N_bian=UAV_lam.*1e6;
NN=length(N_bian);

C_BS_a_1=NaN(1,NN);

C_UAV_a_1=NaN(1,NN);

C_NU_a_1=NaN(1,NN);

C_UU_a_1=NaN(1,NN);

C_BS_a_2=NaN(1,NN);

C_UAV_a_2=NaN(1,NN);

C_NU_a_2=NaN(1,NN);

C_UU_a_2=NaN(1,NN);
C_BS_a_3=NaN(1,NN);

C_UAV_a_3=NaN(1,NN);

C_NU_a_3=NaN(1,NN);

C_UU_a_3=NaN(1,NN);

h=0.01;
parfor i=2:NN

BS_lam=1e-6;

[C_BS_a_1(i)]=C_HPPP_two_BS_T_ana(UE_lam,h,sig2,N_cha,..
.

m_l,m_nl,a_l,a_nl,K_l,K_nl,...

B_BS,BS_lam,P_BS_UE,...
```

```
B_UAV,UAV_lam(i),P_UAV_UE,H_UAV_UE);

[C_UAV_a_1(i)]=C_HPPP_two_UAV_T_ana(UE_lam,h,sig2,N_cha,
...
m_l,m_nl,a_l,a_nl,K_l,K_nl,...
B_BS,BS_lam,P_BS_UE,...
B_UAV,UAV_lam(i),P_UAV_UE,H_UAV_UE);


[C_NU_a_1(i)]=C_NU_HPPP_two_BS_T_ana(UE_lam,h,sig2,N_ch
a,...
m_l,m_nl,a_l,a_nl,K_l,K_nl,...
B_BS,BS_lam,P_BS_UE,...
B_UAV,UAV_lam(i),P_UAV_UE,H_UAV_UE);
C_UU_a_1(i)=C_BS_a_1(i)+C_UAV_a_1(i);
BS_lam=3e-6;
[C_BS_a_2(i)]=C_HPPP_two_BS_T_ana(UE_lam,h,sig2,N_cha,..
.
m_l,m_nl,a_l,a_nl,K_l,K_nl,...
B_BS,BS_lam,P_BS_UE,...
B_UAV,UAV_lam(i),P_UAV_UE,H_UAV_UE);

[C_UAV_a_2(i)]=C_HPPP_two_UAV_T_ana(UE_lam,h,sig2,N_cha,
...
```

```
m_l,m_nl,a_l,a_nl,K_l,K_nl,...

B_BS,BS_lam,P_BS_UE,...

B_UAV,UAV_lam(i),P_UAV_UE,H_UAV_UE);


[C_NU_a_2(i)]=C_NU_HPPP_two_BS_T_ana(UE_lam,h,sig2,N_ch
a,...

m_l,m_nl,a_l,a_nl,K_l,K_nl,...

B_BS,BS_lam,P_BS_UE,...

B_UAV,UAV_lam(i),P_UAV_UE,H_UAV_UE);


C_UU_a_2(i)=C_BS_a_2(i)+C_UAV_a_2(i);



BS_lam=5e-6;



[C_BS_a_3(i)]=C_HPPP_two_BS_T_ana(UE_lam,h,sig2,N_cha,..
.

m_l,m_nl,a_l,a_nl,K_l,K_nl,...

B_BS,BS_lam,P_BS_UE,...

B_UAV,UAV_lam(i),P_UAV_UE,H_UAV_UE);


[C_UAV_a_3(i)]=C_HPPP_two_UAV_T_ana(UE_lam,h,sig2,N_cha,
...

m_l,m_nl,a_l,a_nl,K_l,K_nl,...
```

```
B_BS,BS_lam,P_BS_UE,...

B_UAV,UAV_lam(i),P_UAV_UE,H_UAV_UE);


[C_NU_a_3(i)]=C_NU_HPPP_two_BS_T_ana(UE_lam,h,sig2,N_ch
a,...

m_l,m_nl,a_l,a_nl,K_l,K_nl,...

B_BS,BS_lam,P_BS_UE,...

B_UAV,UAV_lam(i),P_UAV_UE,H_UAV_UE);

C_UU_a_3(i)=C_BS_a_3(i)+C_UAV_a_3(i);

i

end

C_BS_a_1(1)=2*C_BS_a_1(2)-C_BS_a_1(3);

C_UAV_a_1(1)=2*C_UAV_a_1(2)-C_UAV_a_1(3);

C_NU_a_1(1)=2*C_NU_a_1(2)-C_NU_a_1(3);
C_UU_a_1(1)=2*C_UU_a_1(2)-C_UU_a_1(3);

C_BS_a_2(1)=2*C_BS_a_2(2)-C_BS_a_2(3);

C_UAV_a_2(1)=2*C_UAV_a_2(2)-C_UAV_a_2(3);

C_NU_a_2(1)=2*C_NU_a_2(2)-C_NU_a_2(3);
C_UU_a_2(1)=2*C_UU_a_2(2)-C_UU_a_2(3);


C_BS_a_3(1)=2*C_BS_a_3(2)-C_BS_a_3(3);

C_UAV_a_3(1)=2*C_UAV_a_3(2)-C_UAV_a_3(3);
```

```
C_NU_a_3(1)=2*C_NU_a_3(2)-C_NU_a_3(3);
C_UU_a_3(1)=2*C_UU_a_3(2)-C_UU_a_3(3);

figure
hold
N=3;
plot(N_bian,C_UU_a_1.*1e6,'r-');
plot(N_bian,C_NU_a_1.*1e6,'r--');
plot(N_bian,C_UU_a_2.*1e6,'b-');
plot(N_bian,C_NU_a_2.*1e6,'b--');
plot(N_bian,C_UU_a_3.*1e6,'k-');
plot(N_bian,C_NU_a_3.*1e6,'k--');
```

Fig.10 Network throughput density vs. User density

```
BS_lam=1e-6;
UAV_lam=3e-6;
UE_lam=10e-6;
B=80;
B_BS=10.^(B./10);
B_UAV=1;
KK_l=10.^(3./10);
KK_nl=10.^(23./10);
m_l=3;
m_nl=1;
a_l=2.5;
a_nl=4;
K_l=1./(KK_l.*(4.*pi.*2.*10.^9./(3.*10.^8)).^a_l);
K_nl=1./(KK_nl.*(4.*pi.*2.*10.^9./(3.*10.^8)).^a_nl);
P_UAV_UE=5;
H_UAV_UE=300;
m_I=m_nl;
a_I=a_nl;
K_I=K_nl;
P_BS_UE=20;
N_cha=5;
sig2=10.^(-114./10);
gam_db=0;
UE_gam=10.^(gam_db./10);
```

```
UE_lam=(0:0.1:50).*1e-6;
N_bian=UE_lam.*1e6;
NN=length(N_bian);
T_BS_a_1=NaN(1,NN);

T_UAV_a_1=NaN(1,NN);

T_NU_a_1=NaN(1,NN);

T_UU_a_1=NaN(1,NN);

T_BS_a_2=NaN(1,NN);

T_UAV_a_2=NaN(1,NN);

T_NU_a_2=NaN(1,NN);

T_UU_a_2=NaN(1,NN);
T_BS_a_3=NaN(1,NN);

T_UAV_a_3=NaN(1,NN);

T_NU_a_3=NaN(1,NN);

T_UU_a_3=NaN(1,NN);


parfor i=2:NN

BS_lam=1e-6;




[T_BS_a_1(i)]=HPPP_two_BS_T_ana(UE_lam(i),UE_gam,sig2,N
_cha,...

m_l,m_nl,a_l,a_nl,K_l,K_nl,...

B_BS,BS_lam,P_BS_UE,...
```

```
B_UAV,UAV_lam,P_UAV_UE,H_UAV_UE);

[T_UAV_a_1(i)]=HPPP_two_UAV_T_ana(UE_lam(i),UE_gam,sig2,
N_cha,...

m_l,m_nl,a_l,a_nl,K_l,K_nl,...

B_BS,BS_lam,P_BS_UE,...

B_UAV,UAV_lam,P_UAV_UE,H_UAV_UE);


[T_NU_a_1(i)]=NU_HPPP_two_BS_T_ana(UE_lam(i),UE_gam,sig
2,N_cha,...

m_l,m_nl,a_l,a_nl,K_l,K_nl,...

B_BS,BS_lam,P_BS_UE,...

B_UAV,UAV_lam,P_UAV_UE,H_UAV_UE);


T_UU_a_1(i)=T_BS_a_1(i)+T_UAV_a_1(i);


BS_lam=3e-6;


[T_BS_a_2(i)]=HPPP_two_BS_T_ana(UE_lam(i),UE_gam,sig2,N
_cha,...

m_l,m_nl,a_l,a_nl,K_l,K_nl,...

B_BS,BS_lam,P_BS_UE,...
```

```
B_UAV,UAV_lam,P_UAV_UE,H_UAV_UE);


[T_UAV_a_2(i)]=HPPP_two_UAV_T_ana(UE_lam(i),UE_gam,sig2,
N_cha,...

m_l,m_nl,a_l,a_nl,K_l,K_nl,...

B_BS,BS_lam,P_BS_UE,...

B_UAV,UAV_lam,P_UAV_UE,H_UAV_UE);



[T_NU_a_2(i)]=NU_HPPP_two_BS_T_ana(UE_lam(i),UE_gam,sig
2,N_cha,...

m_l,m_nl,a_l,a_nl,K_l,K_nl,...

B_BS,BS_lam,P_BS_UE,...

B_UAV,UAV_lam,P_UAV_UE,H_UAV_UE);


T_UU_a_2(i)=T_BS_a_2(i)+T_UAV_a_2(i);



BS_lam=5e-6;



[T_BS_a_3(i)]=HPPP_two_BS_T_ana(UE_lam(i),UE_gam,sig2,N
_cha,...

m_l,m_nl,a_l,a_nl,K_l,K_nl,...

B_BS,BS_lam,P_BS_UE,...

B_UAV,UAV_lam,P_UAV_UE,H_UAV_UE);
```

```
[T_UAV_a_3(i)]=HPPP_two_UAV_T_ana(UE_lam(i),UE_gam,sig2,
N_cha,...

m_l,m_nl,a_l,a_nl,K_l,K_nl,...

B_BS,BS_lam,P_BS_UE,...

B_UAV,UAV_lam,P_UAV_UE,H_UAV_UE);



[T_NU_a_3(i)]=NU_HPPP_two_BS_T_ana(UE_lam(i),UE_gam,sig
2,N_cha,...

m_l,m_nl,a_l,a_nl,K_l,K_nl,...

B_BS,BS_lam,P_BS_UE,...

B_UAV,UAV_lam,P_UAV_UE,H_UAV_UE);

T_UU_a_3(i)=T_BS_a_3(i)+T_UAV_a_3(i);

i
end

T_BS_a_1(1)=2*T_BS_a_1(2)-T_BS_a_1(3);

T_UAV_a_1(1)=2*T_UAV_a_1(2)-T_UAV_a_1(3);

T_NU_a_1(1)=2*T_NU_a_1(2)-T_NU_a_1(3);
T_UU_a_1(1)=2*T_UU_a_1(2)-T_UU_a_1(3);

T_BS_a_2(1)=2*T_BS_a_2(2)-T_BS_a_2(3);

T_UAV_a_2(1)=2*T_UAV_a_2(2)-T_UAV_a_2(3);

T_NU_a_2(1)=2*T_NU_a_2(2)-T_NU_a_2(3);
T_UU_a_2(1)=2*T_UU_a_2(2)-T_UU_a_2(3);
```

```
T_BS_a_3(1)=2*T_BS_a_3(2)-T_BS_a_3(3);

T_UAV_a_3(1)=2*T_UAV_a_3(2)-T_UAV_a_3(3);

T_NU_a_3(1)=2*T_NU_a_3(2)-T_NU_a_3(3);
T_UU_a_3(1)=2*T_UU_a_3(2)-T_UU_a_3(3);

figure
hold
N=3;
plot(N_bian,T_UU_a_1.*1e6,'r-');
plot(N_bian,T_NU_a_1.*1e6,'r--');
plot(N_bian,T_UU_a_2.*1e6,'b-');
plot(N_bian,T_NU_a_2.*1e6,'b--');
plot(N_bian,T_UU_a_3.*1e6,'k-');
plot(N_bian,T_NU_a_3.*1e6,'k--');
```

Fig.11 Network traversal rate density vs. User density

```
BS_lam=1e-6;
UAV_lam=3e-6;
UE_lam=10e-6;
B=80;
B_BS=10.^(B./10);
B_UAV=1;
KK_l=10.^(3./10);
KK_nl=10.^(23./10);
m_l=3;
m_nl=1;
a_l=2.5;
a_nl=4;
K_l=1./(KK_l.*(4.*pi.*2.*10.^9./(3.*10.^8)).^a_l);
K_nl=1./(KK_nl.*(4.*pi.*2.*10.^9./(3.*10.^8)).^a_nl);
P_UAV_UE=5;
H_UAV_UE=300;

m_I=m_nl;
```

```
a_I=a_nl;

K_I=K_nl;

P_BS_UE=20;

N_cha=5;

sig2=10.^(-114./10);

gam_db=0;
UE_gam=10.^(gam_db./10);
UE_lam=(0:0.5:50).*1e-6;

N_bian=UE_lam.*1e6;
NN=length(N_bian);


C_BS_a_1=NaN(1,NN);

C_UAV_a_1=NaN(1,NN);

C_NU_a_1=NaN(1,NN);

C_UU_a_1=NaN(1,NN);

C_BS_a_2=NaN(1,NN);

C_UAV_a_2=NaN(1,NN);

C_NU_a_2=NaN(1,NN);

C_UU_a_2=NaN(1,NN);
C_BS_a_3=NaN(1,NN);

C_UAV_a_3=NaN(1,NN);

C_NU_a_3=NaN(1,NN);

C_UU_a_3=NaN(1,NN);
```

```
h=0.01;
parfor i=2:NN

BS_lam=1e-6;




[C_BS_a_1(i)]=C_HPPP_two_BS_T_ana(UE_lam(i),h,sig2,N_ch
a,...

m_l,m_nl,a_l,a_nl,K_l,K_nl,...

B_BS,BS_lam,P_BS_UE,...

B_UAV,UAV_lam,P_UAV_UE,H_UAV_UE);


[C_UAV_a_1(i)]=C_HPPP_two_UAV_T_ana(UE_lam(i),h,sig2,N_
cha,...

m_l,m_nl,a_l,a_nl,K_l,K_nl,...

B_BS,BS_lam,P_BS_UE,...

B_UAV,UAV_lam,P_UAV_UE,H_UAV_UE);



[C_NU_a_1(i)]=C_NU_HPPP_two_BS_T_ana(UE_lam(i),h,sig2,N
_cha,...

m_l,m_nl,a_l,a_nl,K_l,K_nl,...

B_BS,BS_lam,P_BS_UE,...

B_UAV,UAV_lam,P_UAV_UE,H_UAV_UE);

C_UU_a_1(i)=C_BS_a_1(i)+C_UAV_a_1(i);

BS_lam=3e-6;
```

```
[C_BS_a_2(i)]=C_HPPP_two_BS_T_ana(UE_lam(i),h,sig2,N_ch
a,...

m_l,m_nl,a_l,a_nl,K_l,K_nl,...

B_BS,BS_lam,P_BS_UE,...

B_UAV,UAV_lam,P_UAV_UE,H_UAV_UE);


[C_UAV_a_2(i)]=C_HPPP_two_UAV_T_ana(UE_lam(i),h,sig2,N_
cha,...

m_l,m_nl,a_l,a_nl,K_l,K_nl,...

B_BS,BS_lam,P_BS_UE,...

B_UAV,UAV_lam,P_UAV_UE,H_UAV_UE);


[C_NU_a_2(i)]=C_NU_HPPP_two_BS_T_ana(UE_lam(i),h,sig2,N
_cha,...

m_l,m_nl,a_l,a_nl,K_l,K_nl,...

B_BS,BS_lam,P_BS_UE,...

B_UAV,UAV_lam,P_UAV_UE,H_UAV_UE);


C_UU_a_2(i)=C_BS_a_2(i)+C_UAV_a_2(i);


BS_lam=5e-6;
```

```
[C_BS_a_3(i)]=C_HPPP_two_BS_T_ana(UE_lam(i),h,sig2,N_ch
a,...

m_l,m_nl,a_l,a_nl,K_l,K_nl,...

B_BS,BS_lam,P_BS_UE,...

B_UAV,UAV_lam,P_UAV_UE,H_UAV_UE);


[C_UAV_a_3(i)]=C_HPPP_two_UAV_T_ana(UE_lam(i),h,sig2,N_
cha,...

m_l,m_nl,a_l,a_nl,K_l,K_nl,...

B_BS,BS_lam,P_BS_UE,...

B_UAV,UAV_lam,P_UAV_UE,H_UAV_UE);



[C_NU_a_3(i)]=C_NU_HPPP_two_BS_T_ana(UE_lam(i),h,sig2,N
_cha,...

m_l,m_nl,a_l,a_nl,K_l,K_nl,...

B_BS,BS_lam,P_BS_UE,...

B_UAV,UAV_lam,P_UAV_UE,H_UAV_UE);

C_UU_a_3(i)=C_BS_a_3(i)+C_UAV_a_3(i);

i

end

C_BS_a_1(1)=2*C_BS_a_1(2)-C_BS_a_1(3);

C_UAV_a_1(1)=2*C_UAV_a_1(2)-C_UAV_a_1(3);

C_NU_a_1(1)=2*C_NU_a_1(2)-C_NU_a_1(3);
```

```
C_UU_a_1(1)=2*C_UU_a_1(2)-C_UU_a_1(3);

C_BS_a_2(1)=2*C_BS_a_2(2)-C_BS_a_2(3);

C_UAV_a_2(1)=2*C_UAV_a_2(2)-C_UAV_a_2(3);

C_NU_a_2(1)=2*C_NU_a_2(2)-C_NU_a_2(3);
C_UU_a_2(1)=2*C_UU_a_2(2)-C_UU_a_2(3);


C_BS_a_3(1)=2*C_BS_a_3(2)-C_BS_a_3(3);

C_UAV_a_3(1)=2*C_UAV_a_3(2)-C_UAV_a_3(3);

C_NU_a_3(1)=2*C_NU_a_3(2)-C_NU_a_3(3);
C_UU_a_3(1)=2*C_UU_a_3(2)-C_UU_a_3(3);

figure
hold
N=3;
plot(N_bian,C_UU_a_1.*1e6,'r-');
plot(N_bian,C_NU_a_1.*1e6,'r--');
plot(N_bian,C_UU_a_2.*1e6,'b-');
plot(N_bian,C_NU_a_2.*1e6,'b--');
plot(N_bian,C_UU_a_3.*1e6,'k-');
plot(N_bian,C_NU_a_3.*1e6,'k--');
```

Fig.12 Network throughput density vs. Number of channel

```
BS_lam=1e-6;
UAV_lam=3e-6;
UE_lam=10e-6;
B=80;
B_BS=10.^(B./10);
B_UAV=1;
KK_l=10.^(3./10);
KK_nl=10.^(23./10);
m_l=3;
m_nl=1;
```

```
a_l=2.5;
a_nl=4;
K_l=1./(KK_l.*(4.*pi.*2.*10.^9./(3.*10.^8)).^a_l);
K_nl=1./(KK_nl.*(4.*pi.*2.*10.^9./(3.*10.^8)).^a_nl);
P_UAV_UE=5;
H_UAV_UE=300;
m_I=m_nl;
a_I=a_nl;
K_I=K_nl;
P_BS_UE=20;
N_cha=5;
sig2=10.^(-114./10);
gam_db=0;
UE_gam=10.^(gam_db./10);
N_cha=0:1:50;

N_bian=N_cha;
NN=length(N_bian);

T_BS_a_1=NaN(1,NN);

T_UAV_a_1=NaN(1,NN);

T_NU_a_1=NaN(1,NN);

T_UU_a_1=NaN(1,NN);

T_BS_a_2=NaN(1,NN);

T_UAV_a_2=NaN(1,NN);

T_NU_a_2=NaN(1,NN);

T_UU_a_2=NaN(1,NN);
T_BS_a_3=NaN(1,NN);

T_UAV_a_3=NaN(1,NN);

T_NU_a_3=NaN(1,NN);

T_UU_a_3=NaN(1,NN);
```

```
parfor i=2:NN

BS_lam=1e-6;



[T_BS_a_1(i)]=HPPP_two_BS_T_ana(UE_lam,UE_gam,sig2,N_ch
a(i),...

m_l,m_nl,a_l,a_nl,K_l,K_nl,...

B_BS,BS_lam,P_BS_UE,...

B_UAV,UAV_lam,P_UAV_UE,H_UAV_UE);


[T_UAV_a_1(i)]=HPPP_two_UAV_T_ana(UE_lam,UE_gam,sig2,N_
cha(i),...

m_l,m_nl,a_l,a_nl,K_l,K_nl,...

B_BS,BS_lam,P_BS_UE,...

B_UAV,UAV_lam,P_UAV_UE,H_UAV_UE);



[T_NU_a_1(i)]=NU_HPPP_two_BS_T_ana(UE_lam,UE_gam,sig2,N
_cha(i),...

m_l,m_nl,a_l,a_nl,K_l,K_nl,...

B_BS,BS_lam,P_BS_UE,...

B_UAV,UAV_lam,P_UAV_UE,H_UAV_UE);

T_UU_a_1(i)=T_BS_a_1(i)+T_UAV_a_1(i);
```

```
BS_lam=3e-6;

[T_BS_a_2(i)]=HPPP_two_BS_T_ana(UE_lam,UE_gam,sig2,N_ch
a(i),...

m_l,m_nl,a_l,a_nl,K_l,K_nl,...

B_BS,BS_lam,P_BS_UE,...

B_UAV,UAV_lam,P_UAV_UE,H_UAV_UE);


[T_UAV_a_2(i)]=HPPP_two_UAV_T_ana(UE_lam,UE_gam,sig2,N_
cha(i),...

m_l,m_nl,a_l,a_nl,K_l,K_nl,...

B_BS,BS_lam,P_BS_UE,...

B_UAV,UAV_lam,P_UAV_UE,H_UAV_UE);

[T_NU_a_2(i)]=NU_HPPP_two_BS_T_ana(UE_lam,UE_gam,sig2,N
_cha(i),...

m_l,m_nl,a_l,a_nl,K_l,K_nl,...

B_BS,BS_lam,P_BS_UE,...

B_UAV,UAV_lam,P_UAV_UE,H_UAV_UE);

T_UU_a_2(i)=T_BS_a_2(i)+T_UAV_a_2(i);

BS_lam=5e-6;

[T_BS_a_3(i)]=HPPP_two_BS_T_ana(UE_lam,UE_gam,sig2,N_ch
a(i),...

m_l,m_nl,a_l,a_nl,K_l,K_nl,...

B_BS,BS_lam,P_BS_UE,...
```

```
B_UAV,UAV_lam,P_UAV_UE,H_UAV_UE);

[T_UAV_a_3(i)]=HPPP_two_UAV_T_ana(UE_lam,UE_gam,sig2,N_
cha(i),...

m_l,m_nl,a_l,a_nl,K_l,K_nl,...

B_BS,BS_lam,P_BS_UE,...

B_UAV,UAV_lam,P_UAV_UE,H_UAV_UE);

[T_NU_a_3(i)]=NU_HPPP_two_BS_T_ana(UE_lam,UE_gam,sig2,N
_cha(i),...

m_l,m_nl,a_l,a_nl,K_l,K_nl,...

B_BS,BS_lam,P_BS_UE,...

B_UAV,UAV_lam,P_UAV_UE,H_UAV_UE);

T_UU_a_3(i)=T_BS_a_3(i)+T_UAV_a_3(i);

I

end

T_BS_a_1(1)=2*T_BS_a_1(2)-T_BS_a_1(3);

T_UAV_a_1(1)=2*T_UAV_a_1(2)-T_UAV_a_1(3);

T_NU_a_1(1)=2*T_NU_a_1(2)-T_NU_a_1(3);
T_UU_a_1(1)=2*T_UU_a_1(2)-T_UU_a_1(3);

T_BS_a_2(1)=2*T_BS_a_2(2)-T_BS_a_2(3);

T_UAV_a_2(1)=2*T_UAV_a_2(2)-T_UAV_a_2(3);

T_NU_a_2(1)=2*T_NU_a_2(2)-T_NU_a_2(3);
T_UU_a_2(1)=2*T_UU_a_2(2)-T_UU_a_2(3);
```

```
T_BS_a_3(1)=2*T_BS_a_3(2)-T_BS_a_3(3);

T_UAV_a_3(1)=2*T_UAV_a_3(2)-T_UAV_a_3(3);

T_NU_a_3(1)=2*T_NU_a_3(2)-T_NU_a_3(3);
T_UU_a_3(1)=2*T_UU_a_3(2)-T_UU_a_3(3);


figure
hold
N=3;
plot(N_bian,T_UU_a_1.*1e6,'r-');
plot(N_bian,T_NU_a_1.*1e6,'r--');
plot(N_bian,T_UU_a_2.*1e6,'b-');
plot(N_bian,T_NU_a_2.*1e6,'b--');
plot(N_bian,T_UU_a_3.*1e6,'k-');
plot(N_bian,T_NU_a_3.*1e6,'k--');
```

Fig.13 Network traversal rate density vs. Number of channel

```
BS_lam=1e-6;
UAV_lam=3e-6;
UE_lam=10e-6;
B=80;
B_BS=10.^(B./10);
B_UAV=1;
KK_l=10.^(3./10);
KK_nl=10.^(23./10);
m_l=3;
m_nl=1;
a_l=2.5;
a_nl=4;
K_l=1./(KK_l.*(4.*pi.*2.*10.^9./(3.*10.^8)).^a_l);
K_nl=1./(KK_nl.*(4.*pi.*2.*10.^9./(3.*10.^8)).^a_nl);
P_UAV_UE=5;
H_UAV_UE=300;
m_I=m_nl;
a_I=a_nl;
```

```
K_I=K_nl;
P_BS_UE=20;
N_cha=5;
sig2=10.^(-114./10);
gam_db=0;
UE_gam=10.^(gam_db./10);
N_cha=0:1:50;



N_bian=N_cha;
NN=length(N_bian);

C_BS_a_1=NaN(1,NN);

C_UAV_a_1=NaN(1,NN);

C_NU_a_1=NaN(1,NN);

C_UU_a_1=NaN(1,NN);

C_BS_a_2=NaN(1,NN);

C_UAV_a_2=NaN(1,NN);

C_NU_a_2=NaN(1,NN);

C_UU_a_2=NaN(1,NN);
C_BS_a_3=NaN(1,NN);

C_UAV_a_3=NaN(1,NN);

C_NU_a_3=NaN(1,NN);

C_UU_a_3=NaN(1,NN);

h=0.01;
parfor i=2:NN

BS_lam=1e-6;
```

```
[C_BS_a_1(i)]=C_HPPP_two_BS_T_ana(UE_lam,h,sig2,N_cha(i)
,...

m_l,m_nl,a_l,a_nl,K_l,K_nl,...

B_BS,BS_lam,P_BS_UE,...

B_UAV,UAV_lam,P_UAV_UE,H_UAV_UE);


[C_UAV_a_1(i)]=C_HPPP_two_UAV_T_ana(UE_lam,h,sig2,N_cha
(i),...

m_l,m_nl,a_l,a_nl,K_l,K_nl,...

B_BS,BS_lam,P_BS_UE,...

B_UAV,UAV_lam,P_UAV_UE,H_UAV_UE);



[C_NU_a_1(i)]=C_NU_HPPP_two_BS_T_ana(UE_lam,h,sig2,N_ch
a(i),...

m_l,m_nl,a_l,a_nl,K_l,K_nl,...

B_BS,BS_lam,P_BS_UE,...

B_UAV,UAV_lam,P_UAV_UE,H_UAV_UE);

C_UU_a_1(i)=C_BS_a_1(i)+C_UAV_a_1(i);

BS_lam=3e-6;

[C_BS_a_2(i)]=C_HPPP_two_BS_T_ana(UE_lam,h,sig2,N_cha(i)
,...

m_l,m_nl,a_l,a_nl,K_l,K_nl,...

B_BS,BS_lam,P_BS_UE,...
```

```
B_UAV,UAV_lam,P_UAV_UE,H_UAV_UE);


[C_UAV_a_2(i)]=C_HPPP_two_UAV_T_ana(UE_lam,h,sig2,N_cha
(i),...

m_l,m_nl,a_l,a_nl,K_l,K_nl,...

B_BS,BS_lam,P_BS_UE,...

B_UAV,UAV_lam,P_UAV_UE,H_UAV_UE);



[C_NU_a_2(i)]=C_NU_HPPP_two_BS_T_ana(UE_lam,h,sig2,N_ch
a(i),...

m_l,m_nl,a_l,a_nl,K_l,K_nl,...

B_BS,BS_lam,P_BS_UE,...

B_UAV,UAV_lam,P_UAV_UE,H_UAV_UE);

C_UU_a_2(i)=C_BS_a_2(i)+C_UAV_a_2(i);

BS_lam=5e-6;

[C_BS_a_3(i)]=C_HPPP_two_BS_T_ana(UE_lam,h,sig2,N_cha(i)
,...

m_l,m_nl,a_l,a_nl,K_l,K_nl,...

B_BS,BS_lam,P_BS_UE,...

B_UAV,UAV_lam,P_UAV_UE,H_UAV_UE);


[C_UAV_a_3(i)]=C_HPPP_two_UAV_T_ana(UE_lam,h,sig2,N_cha
(i),...
```

```
m_l,m_nl,a_l,a_nl,K_l,K_nl,...

B_BS,BS_lam,P_BS_UE,...

B_UAV,UAV_lam,P_UAV_UE,H_UAV_UE);



[C_NU_a_3(i)]=C_NU_HPPP_two_BS_T_ana(UE_lam,h,sig2,N_ch
a(i),...

m_l,m_nl,a_l,a_nl,K_l,K_nl,...

B_BS,BS_lam,P_BS_UE,...

B_UAV,UAV_lam,P_UAV_UE,H_UAV_UE);

C_UU_a_3(i)=C_BS_a_3(i)+C_UAV_a_3(i);

i

end

C_BS_a_1(1)=2*C_BS_a_1(2)-C_BS_a_1(3);

C_UAV_a_1(1)=2*C_UAV_a_1(2)-C_UAV_a_1(3);

C_NU_a_1(1)=2*C_NU_a_1(2)-C_NU_a_1(3);
C_UU_a_1(1)=2*C_UU_a_1(2)-C_UU_a_1(3);

C_BS_a_2(1)=2*C_BS_a_2(2)-C_BS_a_2(3);

C_UAV_a_2(1)=2*C_UAV_a_2(2)-C_UAV_a_2(3);

C_NU_a_2(1)=2*C_NU_a_2(2)-C_NU_a_2(3);
C_UU_a_2(1)=2*C_UU_a_2(2)-C_UU_a_2(3);


C_BS_a_3(1)=2*C_BS_a_3(2)-C_BS_a_3(3);
```

```
C_UAV_a_3(1)=2*C_UAV_a_3(2)-C_UAV_a_3(3);

C_NU_a_3(1)=2*C_NU_a_3(2)-C_NU_a_3(3);
C_UU_a_3(1)=2*C_UU_a_3(2)-C_UU_a_3(3);

figure
hold
N=3;
plot(N_bian,C_UU_a_1.*1e6,'r-');
plot(N_bian,C_NU_a_1.*1e6,'r--');
plot(N_bian,C_UU_a_2.*1e6,'b-');
plot(N_bian,C_NU_a_2.*1e6,'b--');
plot(N_bian,C_UU_a_3.*1e6,'k-');
plot(N_bian,C_NU_a_3.*1e6,'k--');
```
